# Alcohol consumption is prevalent among Chinese adolescents: a national survey

**DOI:** 10.1007/s12519-025-00994-4

**Published:** 2025-12-26

**Authors:** Fu-Lin Huang, Ning Ji, Yi-Meng Mao, Xin-Ying Zeng, Ji Tang, Sally Casswell, Sheng-Gen Wu, Shi-Wei Liu

**Affiliations:** 1 Emergency Response and Epidemic Management Institute, Fujian Provincial Center for Disease Control and Prevention, Chong’an Road, Jin’an District, Fuzhou, 350012 China; 2 National Center for Chronic and Noncommunicable Disease Control and Prevention, Chinese Center for Disease Control and Prevention, Beijing, 100050 China; 3 Health Education and Promotion Institute, Fujian Provincial Center for Disease Control and Prevention, Fuzhou, 350012 China; 4 Tobacco Control Office, Chinese Center for Disease Control and Prevention, 27# Nanwei Road, Xicheng District, Beijing, 100050 China; 5 Chinese Preventive Medicine Association, Beijing, 100050 China; 6 Social and Health Outcomes Research and Evaluation (SHORE)SHORE & Whariki Research CentreCollege of Health, Massey University, Auckland, New Zealand

**Keywords:** Adolescents, Alcohol use, China, Cross-sectional study, Drinking pattern, Public health

## Abstract

**Background:**

The consumption of alcohol by adolescents has deleterious effects on their health and cognitive functions. Adolescent alcohol consumption represents a significant public health issue. Up-to-date national surveys examining alcohol use among Chinese adolescents is lacking. This study aims to offer nationally representative insights into the prevalence and patterns of alcohol consumption among Chinese adolescents.

**Methods:**

A school-based, nationally representative cross-sectional survey targeting middle and high school students (aged 12– < 19 years) was conducted using a multi-stage stratified cluster random sampling design in 2021. Self-reported questionnaires were used to collect data on the prevalence of drinking and drunkenness over lifetime, past year, and past month, early onset of drinking and drunkenness, alcoholic beverage types, drinking frequency, emotional motives during drinking episodes and drinking occasions and locations. Estimates were weighted for the complex sampling design.

**Results:**

The survey revealed that 44.1%, 32.7%, and 11.2% had consumed alcohol in their lifetime, past year and past month respectively. Prevalence of drunkenness for the same periods was 12.1%, 5.9%, and 1.6%. Totally 31.2% of students reported alcohol use at age 13 or younger and 6.8% reported early drunkenness. The most consumed alcoholic beverages among past-year drinkers were beer (71.1%) followed by wine (69.4%). Adolescent drinking is often passive without emotional motives (36.0%), or motivated by joy (31.3%) or sadness (23.6%). Adolescent drinking primarily occurs during family gatherings (51.0%), with private homes being the most common drinking location (68.9%).

**Conclusions:**

Alcohol consumption is prevalent among Chinese adolescents, increasing with school grade; percentages of drunkenness are relatively lower. Drinking and drunkenness in some time frames has significantly decreased. Of note, boys demonstrate higher percentages across almost all patterns of alcohol use. Adolescents display disparities in alcohol consumption based on their urban–rural residence and geographical location.

**Graphical abstract:**

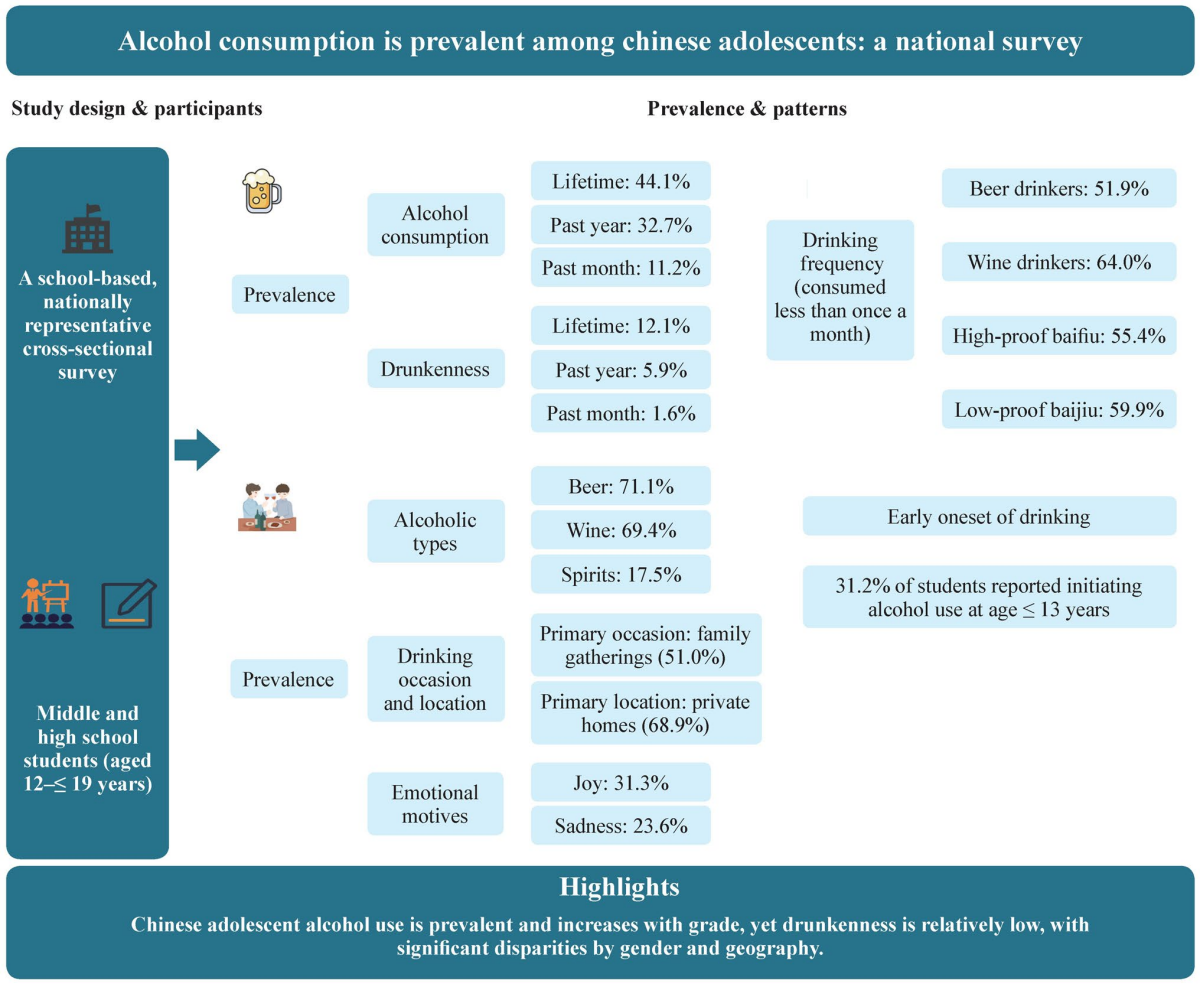

**Supplementary Information:**

The online version contains supplementary material available at 10.1007/s12519-025-00994-4.

## Introduction

The consumption of alcohol by adolescents, which occurs during a critical period of neurodevelopmental vulnerability, has deleterious effects on their health. This includes development of acute liver disease, involvement in traffic accidents, engagement in risky sexual behaviors, participation in violent activities and an increased risk of suicide. Moreover, alcohol consumption can also lead to cognitive decline and impair learning abilities [1]. The consumption of alcohol has emerged as a significant global public health concern, including within China. The projected per capita alcohol consumption among adults (≥ 15 years) worldwide is expected to increase from 5.9 L in 1990 to 7.6 L in 2030, while the prevalence of drinking in past year is anticipated to rise from 45% to 50% [2]. The China Chronic Disease and Nutrition Surveillance System (CCDNS) report indicates that in 2004, 46.5% of adults aged 18–69 reported alcohol consumption in the previous 12 months, with males at 69.3% and females at 22.3% [3]. By 2013, the percentage of adults aged 18 and above who consumed alcohol had decreased to 37.1% (males at 58.3%, females at 15.4%) [4]. However, by 2018, this figure had slightly increased to 39.8% (males at 60.3%, females at 19.1%) [5]. In summary, while alcohol consumption among Chinese adults has generally declined, there has been a slight upward trend in recent years. According to the Global Burden of Disease Study 2021, alcohol consumption in China accounts for 3.25% of deaths and 3.27% of disability-adjusted life years (DALYs), ranking ninth among a spectrum of 88 evaluated risk factors; this exceeds the global average. Furthermore, alcohol consumption emerged as the primary contributing factor to DALYs among individuals aged 5–14, accounting for 0.51% [6]. Given that alcohol consumption often begins during early life and that adolescent alcohol consumption is associated with subsequent alcohol use in adulthood along with the development of long-term health complications, it is crucial to address teenage drinking to effectively tackle the burden of alcohol-related diseases. Several surveys pertaining to alcohol use have been performed in China. A 2005 survey covering 18 provinces with 181,832 students in grades 7–12 showed that the lifetime and past month prevalence of alcohol use was 64.0% and 24.8%, respectively and the prevalence of drunkenness in the past year was 14.6% [7]. A 2008 survey involving 369,337 middle and high school students from 28 provinces indicated that 29.9% had drunk alcohol in the past month [8]. Furthermore, several regional surveys have been conducted. In 2013–2014, a study encompassing 27,762 adolescents and young people aged 12–20 years across six cities revealed that the lifetime, past year and past month prevalence of alcohol use was 56.6%, 44.8%, and 23.3%, respectively; there was a lifetime prevalence of drunkenness of 19.3% [9]. There has been a lack of nationally representative surveys examining alcohol use among Chinese adolescents since 2008. This study aims to address this gap by providing nationally representative insights into the prevalence and habits of alcohol use among Chinese adolescents.

## Methods

### Study design and participants

The survey received ethical approval from the Ethical Review Committee of Chinese Center for Disease Control and Prevention (No. 202115). Informed consent was duly obtained from both school authorities and individual participants.

The target population of this study comprises all middle and high school students in grades 7–12, aged 12–< 19, in the Mainland of China. The typical age ranges for students in grades 7–12 were as follows: 12–< 14 years old for 7th, 13–< 15 for 8th, 14–< 16 for 9th, 15–< 17 for 10th, 16–< 18 for 11th and 17–< 19 for 12th. The survey was carried out in 2021 using a multi-stage stratified cluster sampling method. Initially, seven provinces were selected, each representing different geographical areas in mainland China. Using proportional-to-size sampling (PPS), five districts and five counties were chosen from each province. Within the selected district or county, three middle schools, two academic high schools and one vocational high school were randomly selected. A total of 420 schools participated in the survey. From every grade in each school, one class was randomly selected and all students in those classes participated in a self-reporting survey. Altogether, 59,748 individuals were involved.

### Measurements

Drinking and drunkenness status were assessed by asking participants, “Have you had at least one drink in the past 12 months? (One drink is equivalent to half a bottle/can of beer, a small cup of white wine, a glass of wine, or a glass of rice wine)” and “Have you ever been drunk in the past 12 months? (Drunkenness: a person experiences symptoms such as confusion, slurred speech, vomiting, coma and so on caused by excessive drinking). Possible choices for both inquiries encompassed “1 = Yes, before the previous 30 days”, “2 = Yes, in the previous 30 days”, “3 = Haven’t had a drink in the previous 12 months.” In addition, for the inquiry on drunkenness status, a fourth option of “4 = Haven’t been drunk in the previous 12 months” was included. Choosing option 2 signifies drinking or drunkenness within the past month, while selecting 1 indicates drinking or drunkenness over the past year.

Early onset of drinking and drunkenness was assessed through inquiring the age at which individuals first consumed alcohol and experienced intoxication. Respondents were given a range of age options to choose from, including 7 and under, 8–< 10, 10–< 12, 12–< 14, 14–< 16, 16–< 18, 18 and older, or “I never drink”. For the question regarding drunkenness, there was also an option stating “I have never been drunk”. Participants who reported initiating alcohol consumption at the age of 13 or younger were categorized as having early onset drinking or early onset drunkenness. In addition, based on responses to these questions, we determined the prevalence of lifetime alcohol drinking and drunkenness. Respondents who provided an age at which they started to drink were classified as lifetime drinkers while those who indicated an age for their first experience with drunkenness were classified as lifetime cases of drunkenness. The prevalence and frequency of consumption of various alcoholic beverages, including beer, grape wine, fruit wine, rice wine, yellow rice wine, high-proof baijiu, low-proof baijiu, qingke spirit and others was assessed. Consumption frequencies were categorized into five groups: ≥ 5 days/week, 3–4 days/week, 1–2 days/week, 1–3 days/month, and < 1 day/month.

Usual emotional motives, occasions and locations associated with drinking among the respondents were further investigated. Eight distinct emotional motives were identified, including feelings of sadness, joy, anxiety, loneliness or anger; drinking as part of a daily routine; or drinking without emotional motives (passive drinking). Drinking occasions were classified into five main types: gathering with friends or classmates, family gathering, banquet (including wedding and funeral), drink alone and other occasions. In addition, drinking locations were categorized into six types: one’s own or someone else’s home, hotel, bar, restaurant, karaoke television (KTV)/music bar and other locations. KTV refers to commercial karaoke entertainment venues where patrons rent private rooms to sing, consume beverages, and socialize.

### Statistical analysis

Considering the complex sampling design, a comprehensive weighted strategy was implemented. The overall weight assigned to each respondent comprised three components: base weight, non-response adjustment weight and post-stratification adjustment weight (Formula 1). Base weight was initially derived through the multiplication of province weight, primary sampling unit (PSU) weight, which inversely corresponds to the sampling probability proportional to population size, school weight, reflecting the reciprocal of the sampling probability adjusted by student numbers and class weight, representing the total number of classes at the given grade. For non-response adjustment, the weight was generated by multiplying the reciprocals of the response rates across province, PSU, school, class and individual levels. Post-stratification adjustment weight was calculated based on the distribution within strata defined by urban-rural status, school type (middle school, academic high school, or vocational high school), sex and grade level. Upon consolidating these weights into the overall weight for each participant, point estimates and corresponding 95% confidence intervals (CIs) were computed for all proportions under examination [10]. The Rao-Scott chi-square test based on univariable regression analysis for complex survey design was used to assess disparities among groups. Statistical significance was determined using a two-tailed *P*-value of < 0.05. All data analyses were conducted utilizing SAS version 9.4.

Formulas:

1 $$Weight = R \, \times \,W_{ps,k}$$

$$R = \,W_{s} \, \times R_{1} \, \times R_{2} \, \times \,R_{3} \, \times R_{4} \, \times R_{5}$$

$${{w}_{s}={{w}_{s0}\times w}_{s1}\times {w}_{s2}\times w}_{s3}$$

${w}_{s}$ *basic weight*, ${w}_{s}$_0_ province weight, ${w}_{s}$_1_ PSU weight, ${w}_{s}$_2_ school weight $,{w}_{s}$_3_ class weight. *R* non-response adjustment rate, *R*_*1*_ non-response adjustment rate for provinces, *R*_*2*_ non-response adjustment rate for primary sampling unit(PSU), *R*_*3*_ non-response adjustment rate for school, *R*_*4*_ non-response adjustment rate for class, *R*_*5*_ non-response adjustment rate for individuals

$${w}_{ps,k}=\frac{\text{The number of students reported at the K}-\text{th level in the specific region }}{\text{The sum of the basic weight of the sample at the K}-\text{th level after non}-\text{response adjustment}}$$

## Results

Of the 59,748 eligible individuals 96.0% (or precisely 57,336) participants were recruited (Supplementary Table 1). Among them, 48.3% were girls and 59.4% resided in urban areas. In addition, middle school students accounted for 51.8% of the total participants; academic high school students comprised 35.9% and vocational high school students constituted 12.3%.

In 2021, estimated prevalence of lifetime, past year, and past month drinking among Chinese adolescents was 44.1%, 32.7% and 11.2% respectively (Table 1). In boys (49.7%, 38.3%, 14.2%) there was a significantly higher prevalence of drinking compared to girls (38.0%, 26.5%, 7.9%). There was no statistically significant difference in lifetime drinking prevalence between urban and rural areas, but the drinking prevalence in the past year and the past month were higher in rural (35.1%, 12.1%) compared to urban areas (28.8%, 9.8%). Incidences were lower in the Northwest (33.8%, 25.5%, 9.5%) and Northeast (33.8%, 25.7%, 9.9%) and highest in the Southwest (46.5%, 35.1%, 11.5%). Incidence of drinking in high school students (55.1%, 41.0%, 14.4%) was higher than middle school students (35.8%, 26.4%, 8.9%), with an upward trend as grade levels increased. Moreover, the incidence of drinking in students who smoked or used e-cigarettes was higher than non-users (Supplementary Tables 2 and 3).

**Table 1:** Prevalence of alcohol drinking over a lifetime, past year, and past month among middle and high school students in China, 2021

Variables	Lifetime drinking			Past year drinking			Past month drinking			
	Unweighted, *n*	Weighted % (95% CI)	OR (95% CI)	Unweighted, *n*	Weighted % (95% CI)	OR (95% CI)	Unweighted, *n*		Weighted % (95% CI)	OR (95% CI)
Total	24,085	44.1 (39.9–48.3)		18,130	32.7 (29.4–36.1)		6232		11.2 (9.8–12.7)	
Gender										
Girls	10,153	38.0 (34.1–42.0)	1.0	7163	26.5 (24.0–29.1)	1.0	2207		7.9 (6.8–9.1)	1.0
Boys	13,932	49.7 (45.2–54.2)	1.6 (1.5–1.7)	10,967	38.3 (34.2–42.5)	1.7 (1.6–1.9)	4025		14.2 (12.6–15.9)	1.9 (1.8–2.0)
Residence										
Urban	13,849	40.0 (38.3–41.8)	1.0	8088	28.8 (27.5–30.0)	1.0	3459		9.8 (9.2–10.4)	1.0
Rural	10,236	46.6 (39.9–53.4)	1.3 (1.0–1.7)	10,042	35.1 (29.6–40.5)	1.3 (1.0–1.7)	2773		12.1 (9.8–14.3)	1.3 (1.0–1.6)
Region										
Northwest	2094	33.8 (24.3–43.3)	1.0	1606	25.5 (18.5–32.4)	1.0	603		9.5 (6.9–12.1)	1.0
Northeast	1899	33.8 (28.8–38.8)	1.0 (0.6–1.6)	1478	25.7 (21.9–29.4)	1.0 (0.7–1.5)	573		9.9 (8.8–11.0)	1.0 (0.8–1.4)
North	3244	39.1 (37.2–41.1)	1.3 (0.8–1.9)	2384	27.7 (26.3–29.2)	1.1 (0.8–1.6)	898		9.6 (8.4–10.8)	1.0 (0.7–1.4)
Central	5066	51.1 (43.2–59.0)	2.0 (1.2–3.5)	4003	40.0 (32.4–47.6)	1.9 (1.2–3.2)	1302		12.9 (9.5–16.3)	1.4 (0.9–2.2)
East	3901	46.1 (33.3–59.0)	1.7 (0.9–3.3)	2715	32.1 (22.4–41.8)	1.4 (0.8–2.5)	898		11.5 (7.5–15.6)	1.2 (0.8–2.0)
South	3589	40.5 (34.9–46.1)	1.3 (0.8–2.2)	2768	31.4 (28.4–34.5)	1.3 (0.9–2.0)	934		10.7 (8.6–12.7)	1.1 (0.8–1.6)
Southwest	4292	46.5 (41.9–51.1)	1.7 (1.1–2.7)	3176	35.1 (31.6–38.7)	1.6 (1.1–2.4)	1024		11.5 (10.1–13.0)	1.2 (0.9–1.7)
School type										
Middle school	9718	35.8 (31.1–40.6)	1.0	7260	26.4 (22.6–30.3)	1.0	2302		8.9 (7.6–10.1)	1.0
High school	14,367	55.1 (51.9–58.3)	2.2 (1.8–2.7)	10,870	41.0 (38.7–43.4)	1.9 (1.6–2.3)	3930		14.4 (12.8–16.0)	1.7 (1.6–1.9)
Academic high school	10,533	54.1 (50.6–57.6)	1.0	7782	39.4 (36.6–42.2)	1.0	2630		12.8 (11.4–14.2)	1.0
Vocational high school	3834	57.4 (51.0–63.8)	1.1 (0.9–1.5)	3088	44.9 (41.0–48.9)	1.3 (1.0–1.5)	1300		18.2 (15.0–21.5)	1.5 (1.2–1.9)
Grade										
7 (12–< 14 y)	2701	29.8 (24.5–35.1)	1.0	1955	21.5 (17.7–25.4)	1.0	554		6.8 (6.1–7.6)	1.0
8 (13–< 15 y)	3244	34.8 (29.4–40.3)	1.3 (1.1–1.5)	2387	24.4 (19.0–29.9)	1.2 (1.0–1.4)	782		8.2 (6.0–10.4)	1.2 (0.9–1.7)
9 (14–< 16 y)	3773	43.3 (38.6–48.1)	1.8 (1.6–2.1)	2918	33.7 (30.4–37.0)	1.9 (1.6–2.1)	966		11.6 (9.5–13.8)	1.8 (1.4–2.3)
10 (15–< 17 y)	4470	52.2 (47.6–56.7)	2.6 (2.1–3.1)	3430	39.4 (36.4–42.4)	2.4 (1.9–2.9)	1145		12.4 (10.8–13.9)	1.9 (1.6–2.3)
11 (16–< 18 y)	4889	54.7 (51.2–58.2)	2.8 (2.1–3.8)	3632	39.7 (35.6–43.8)	2.4 (1.9–3.1)	1335		14.8 (13.3–16.3)	2.4 (2.0–2.7)
12 (17–< 19 y)	5008	59.0 (54.0–64.0)	3.4 (2.6–4.5)	3808	44.4 (40.0–48.9)	2.9 (2.2–3.8)	1450		16.4 (13.4–19.5)	2.7 (2.1–3.4)
Cigarette use										
No	22,150	42.3 (38.3–46.4)	1.0	16,339	30.8 (27.7–33.9)	1.0	5209		9.9 (8.6–11.1)	1.0
Yes	1921	91.4 (89.5–93.3)	14.5 (11.3–18.6)	1779	82.6 (79.7–85.5)	10.7 (8.9–12.8)	1018		47.0 (42.9–51.1)	8.1 (6.9–9.5)
E-cigarette use										
No	22,506	42.9 (38.8–47.0)	1.0	16,679	31.4 (28.2–34.6)	1.0	5423		10.3 (9.0–11.6)	1.0
Yes	1439	91.9 (87.8–96.1)	15.2 (9.8–23.5)	1338	83.0 (78.2–87.9)	10.7 (8.5–13.4)	763		47.3 (42.3–52.3)	7.8 (6.6–9.3)

Monitoring points in counties and county-level cities are defined as rural areas, while monitoring points in districts are defined as urban areas

*CI* confidence interval, *OR* odds ratio

Estimated prevalence of lifetime, past year and past month drunkenness was 12.1%, 5.9% and 1.6% respectively (Table 2). Percentages were higher in boys (15.4%, 7.7%, 2.1%) than girls (8.7%, 3.8%, 1.0%), rural areas (13.9%, 6.8%, 1.8%) than urban areas (9.3%, 4.3%, 1.2%), high schools (15.4%, 8.1%, 1.9%) than middle schools (9.2%, 4.2%, 1.3%) and vocational high schools (20.5%, 12.3%, 3.3%) than academic high schools (13.3%, 6.3%, 1.4%). Figures were lowest in North China (6.7%, 3.2%, 0.9%) and higher in Central (15.0%, 6.9%, 2.1%) and Southwest China (15.9%, 7.6%, 2.0%). Ninth grade middle school students reported higher lifetime and past year drunkenness compared to 7th graders, but there is no noticeable difference in the past 30 days. No significant variations in drunkenness were observed across different grades in high school. In addition, students who smoked or used e-cigarettes experienced higher levels of drunkenness than students who did not (Supplementary Tables 4 and 5).

**Table 2:** Prevalence of alcohol drunkenness over a lifetime, past year, and past month among middle and high school students in China, 2021

Variables	Lifetime drunkenness			Past year drunkenness			Past month drunkenness		
	Unweighted, *n*	Weighted % (95% CI)	OR (95% CI)	Unweighted, *n*	Weighted % (95% CI)	OR (95% CI)	Unweighted, *n*	Weighted % (95% CI)	OR (95% CI)
Total	6225	12.1 (10.2–14.1)		3171	5.9 (4.8–6.9)		878	1.6 (1.3–1.9)	
Gender									
Girls	2130	8.7 (6.7–10.6)	1.0	972	3.8 (2.7–4.9)	1.0	265	1.0 (0.7–1.2)	1.0
Boys	4095	15.4 (13.3–17.4)	1.9 (1.6–2.2)	2199	7.7 (6.5–9.0)	2.1 (1.7–2.7)	613	2.1 (1.7–2.6)	2.2 (1.9–2.6)
Residence									
Urban	3190	9.3 (8.3–10.2)	1.0	1582	4.3 (3.8–4.9)	1.0	421	1.2 (0.9–1.4)	1.0
Rural	3035	13.9 (10.8–17.0)	1.6 (1.2–2.1)	1589	6.8 (5.0–8.5)	1.6 (1.2–2.2)	457	1.8 (1.4–2.3)	1.6 (1.1–2.2)
Region									
North	665	6.7 (5.5–8.0)	1.0	354	3.2 (2.2–4.3)	1.0	92	0.9 (0.4–1.3)	1.0
Northeast	615	11.1 (8.2–13.9)	1.7 (1.2–2.5)	382	6.5 (4.5–8.5)	2.1 (1.3–3.3)	94	1.6 (1.3–2.0)	1.9 (1.1–3.4)
Northwest	669	11.4 (7.1–15.7)	1.8 (1.1–2.9)	376	6.1 (4.0–8.2)	1.9 (1.2–3.2)	121	2.0 (1.0–3.0)	2.4 (1.2–4.9)
Central	1376	15.0 (12.1–17.9)	2.4 (1.8–3.3)	668	6.9 (5.3–8.5)	2.2 (1.5–3.3)	193	2.1 (1.0–3.1)	2.5 (1.2–5.0)
East	776	11.7 (6.4–16.9)	1.8 (1.1–3.2)	354	5.5 (2.5–8.4)	1.7 (0.9–3.3)	93	1.3 (0.9–1.7)	1.5 (0.8–2.8)
South	827	9.6 (7.6–11.5)	1.5 (1.1–2.0)	417	4.8 (3.6–5.9)	1.5 (1.0–2.3)	121	1.3 (0.9–1.8)	1.6 (0.9–2.9)
Southwest	1297	15.9 (11.2–20.7)	2.6 (1.8–4.0)	620	7.6 (4.8–10.4)	2.4 (1.5–4.1)	164	2.0 (1.0–3.0)	2.4 (1.2–4.8)
School type									
Middle school	2415	9.2 (7.2–11.1)	1.0	1130	4.2 (3.2–5.1)	1.0	329	1.3 (1.0–1.7)	1.0
High school	3810	15.4 (13.7–17.2)	1.8 (1.5–2.2)	2041	8.1 (6.8–9.3)	2.0 (1.7–2.4)	549	1.9 (1.6–2.3)	1.5 (1.1–1.9)
Academic high school	2340	13.3 (11.1–15.6)	1.0	1183	6.3 (5.0–7.6)	1.0	288	1.4 (1.0–1.7)	1.0
Vocational high school	1470	20.5 (18.3–22.8)	1.7 (1.3–2.1)	858	12.3 (10.5–14.1)	2.1 (1.6–2.7)	261	3.3 (2.3–4.3)	2.5 (1.6–3.8)
Grade									
7 (12–< 14 y)	659	8.0 (6.6–9.5)	1.0	297	3.5 (2.7–4.3)	1.0	79	1.0 (0.5–1.5)	1.0
8 (13–< 15 y)	760	8.3 (6.0–10.7)	1.0 (0.8–1.3)	367	3.8 (2.5–5.1)	1.1 (0.7–1.6)	112	1.4 (0.7–2.1)	1.4 (0.6–3.2)
9 (14–< 16 y)	996	11.9(9.0–14.8)	1.5 (1.2–1.9)	466	5.3 (3.9–6.7)	1.6 (1.2–2.0)	138	1.5 (0.8–2.2)	1.4 (0.7–3.0)
10 (15–< 17 y)	1113	15.1 (10.9–19.4)	2.1 (1.6–2.7)	577	8.1 (4.7–11.5)	2.4 (1.5–4.1)	159	1.8 (1.3–2.3)	1.7 (1.0–3.0)
11 (16–< 18 y)	1272	15.0 (11.9–18.2)	2.0 (1.5–2.7)	697	7.5 (4.9–10.1)	2.3 (1.5–3.4)	180	1.7 (1.2–2.2)	1.7 (1.0–2.8)
12 (17–< 19 y)	1425	17.1 (14.8–19.4)	2.3 (1.9–2.8)	767	8.6 (7.0–10.3)	2.6 (2.0–3.4)	210	2.3 (1.7–2.9)	2.2 (1.4–3.6)
Cigarette use									
No	5106	10.5 (8.8–12.2)	1.0	2345	4.6 (3.7–5.5)	1.0	555	1.1 (0.9–1.3)	1.0
Yes	1115	57.0 (52.6–61.4)	11.2 (9.1–13.8)	2122	39.0 (34.5–43.5)	13.3 (10.3–17.1)	321	14.8 (11.6–18.0)	15.9 (11.5–21.8)
E-cigarette use									
No	5409	11.1 (9.3–12.9)	1.0	2561	5.0 (4.0–6.0)	1.0	2561	5.0 (4.0–6.0)	1.0
Yes	752	52.8 (48.0–57.6)	9.1 (8.0–10.2)	566	35.9 (31.4–40.4)	10.6 (8.1–13.9)	566	35.9 (31.4–40.4)	12.4 (8.8–17.3)

Monitoring points in counties and county-level cities are defined as rural areas, while monitoring points in districts are defined as urban areas

*CI* confidence interval, *OR* odds ratio

As shown in Table 3, 31.2% of students reported alcohol use at age 13 or younger. Boys (35.9%) were more likely than girls (26.1%) to engage in early alcohol use, with no statistically significant differences observed between urban and rural areas, as well as between middle and high school students. The highest percentage of students reporting early alcohol use was found in Central China (37.6%), the lowest was in Northwest China (18.8%). Totally 6.8% of students reported early onset of drunkenness at age of 13 or younger, with a higher prevalence in boys (8.5%) than girls (5.0%), and in rural areas (8.0%) compared to urban areas (4.9%). There were no statistically significant differences between middle and high school students. The highest incidence of students reporting early drunkenness was in Central China (8.9%); the lowest was in North China (3.9%) (Table 3).

**Table 3:** Proportion of middle and high school students who started drinking alcohol and experienced drunkenness by the age of 13 in China, 2021

Variables	Onset age % (95% CI)							
	Drink alcohol				Get drunk on alcohol			
	Among all students		Among lifetime drinkers		Among all students		Among students who have experienced being drunk	
	≤ 13 y	> 13 y	≤ 13 y	> 13 y	≤ 13 y	> 13 y	≤ 13 y	> 13 y
Total	31.2 (28.0–34.5)	12.9 (11.6–14.3)	70.8 (68.8–72.7)	29.2 (27.3–31.2)	6.8 (5.5–8.1)	5.3 (4.4–6.2)	56.1 (51.9–60.4)	43.9 (39.6–48.1)
Gender								
Girls	26.1 (22.7–29.4)	12.0 (10.7–13.3)	68.5 (65.5–71.6)	31.5 (28.4–34.5)	5.0 (3.4–6.6)	3.7 (3.0–4.4)	55.4 (51.9–58.9)	44.6 (41.1–48.1)
Boys	35.9 (32.7–39.2)	13.8 (12.0–15.5)	72.3 (70.3–74.4)	27.7 (25.6–29.7)	8.5 (7.3–9.7)	6.9 (5.7–8.0)	57.6 (50.1–65.2)	42.4 (34.8–49.9)
Residence								
Urban	28.6 (26.6–30.5)	11.5 (10.4–12.5)	71.4 (68.6–74.1)	28.6 (25.9–31.4)	4.9 (4.2–5.6)	4.4 (3.8–5.0)	52.8 (48.5–57.1)	47.2 (42.9–51.5)
Rural	32.8 (27.7–38.0)	13.8 (11.7–15.9)	70.4 (67.8–73.1)	29.6 (26.9–32.2)	8.0 (5.9–10.1)	5.9 (4.5–7.3)	57.5 (51.9–63.1)	42.5 (36.9–48.1)
Region								
Northwest	18.8 (15.0–22.6)	15.0 (12.9–17.2)	55.6 (50.7–60.5)	44.4 (39.5–49.3)	4.9 (3.5–6.3)	6.2 (4.3–8.0)	44.3 (37.5–51.1)	55.7 (48.9–62.5)
Northeast	20.7 (15.6–25.8)	13.1 (7.9–18.3)	61.3 (54.3–68.2)	38.7 (31.8–45.7)	4.8 (3.5–6.1)	6.6 (3.5–9.8)	41.8 (35.0–48.6)	58.2 (51.4–65.0)
North	30.7 (28.9–32.5)	8.5 (8.1–8.8)	78.4 (77.4–79.4)	21.6 (20.6–22.6)	3.9 (3.3–4.6)	2.8 (2.1–3.5)	58.6 (53.9–63.3)	41.4 (36.7–46.1)
Central	37.6 (32.2–43.1)	13.4 (10.4–16.5)	73.7 (70.7–76.7)	26.3 (23.3–29.3)	8.9 (7.0–10.8)	6.1 (4.6–7.5)	59.4 (54.0–64.8)	40.6 (35.2–46.0)
East	33.5 (23.2–43.7)	12.7 (9.8–15.6)	72.5 (69.2–75.8)	27.5 (24.2–30.8)	7.5 (3.6–11.3)	4.2 (2.4–6.0)	64.0 (55.0–72.9)	36.0 (27.1–45.0)
South	26.9 (21.5–32.2)	13.6 (12.0–15.3)	66.4 (61.1–71.6)	33.6 (28.4–38.9)	4.7 (3.3–6.1)	4.9 (3.8–6.0)	49.2 (41.1–57.3)	50.8 (42.7–58.9)
Southwest	32.1 (28.8–35.5)	14.4 (10.1–18.6)	69.1 (61.9–76.4)	30.9 (23.6–38.1)	8.3 (5.4–11.2)	7.6 (4.6–10.7)	52.2 (40.8–63.6)	47.8 (36.4–59.2)
School type								
Middle school	31.2 (27.3–35.2)	4.6 (3.4–5.8)	87.2 (84.8–89.5)	12.8 (10.5–15.2)	7.1 (5.8–8.4)	2.2 (1.4–3.1)	76.2 (71.5–80.8)	23.8 (19.2–28.5)
High school	31.2 (28.1–34.3)	23.9 (22.0–25.7)	56.7 (53.2–60.1)	43.3 (39.9–46.8)	6.4 (4.7–8.1)	9.4 (8.4–10.4)	40.4 (33.2–47.6)	59.6 (52.4–66.8)
Academic high school	32.0 (29.4–34.7)	22.1 (19.5–24.7)	59.2 (55.6–62.8)	40.8 (37.2–44.4)	6.1 (4.2–8.1)	7.6 (6.2–8.9)	44.6 (35.2–54.0)	55.4 (46.0–64.8)
Vocational high school	29.2 (23.2–35.3)	28.2 (26.1–30.2)	50.9 (45.5–56.4)	49.1 (43.6–54.5)	7.1 (5.5–8.7)	14.0 (12.1–15.9)	33.8 (27.9–39.7)	66.2 (60.3–72.1)
Grade								
7 (12– < 14 y)	28.6 (24.1–33.1)	1.2 (0.3–2.1)	96.0 (93.5–98.4)	4.0 (1.6–6.5)	7.5 (6.1–8.9)	0.5 (0.1–0.9)	93.4 (88.9–97.9)	6.6 (2.1–11.1)
8 (13– < 15 y)	31.9 (27.0–36.8)	2.9 (1.6–4.3)	91.6 (88.2–95.1)	8.4 (4.9–11.8)	6.8 (4.8–8.9)	1.5 (0.8–2.2)	82.2 (75.0–89.4)	17.8 (10.6–25.0)
9 (14– < 16 y)	33.4 (29.7–37.0)	10.0 (8.4–11.6)	77.0 (74.6–79.3)	23.0 (20.7–25.4)	7.1 (5.7–8.4)	4.8 (3.1–6.6)	59.3 (53.5–65.2)	40.7 (34.8–46.5)
10 (15– < 17 y)	34.2 (29.4–39.0)	17.9 (16.0–19.9)	65.6 (61.0–70.2)	34.4 (29.8–39.0)	8.5 (4.1–13.0)	7.0 (6.1–7.8)	55.1 (41.5–68.7)	44.9 (31.3–58.5)
11 (16– < 18 y)	30.1 (27.6–32.6)	24.6 (22.2–27.0)	55.0 (51.9–58.2)	45.0 (41.9–48.1)	5.4 (4.2–6.7)	9.6 (7.5–11.8)	36.1 (32.0–40.1)	63.9 (59.9–68.0)
12 (17– < 19 y)	28.9 (25.2–32.5)	30.1 (27.8–32.5)	48.9 (45.8–52.0)	51.1 (48.0–54.2)	4.9 (4.2–5.6)	12.2 (10.3–14.0)	28.7 (25.5–31.8)	71.3 (68.2–74.5)
Cigarette use								
No	62.1 (57.6–66.6)	29.3 (24.7–33.9)	68.0 (63.1–72.9)	32.0 (27.1–36.9)	22.8 (19.3–26.4)	34.2 (30.9–37.5)	40.0 (35.3–44.8)	60.0 (55.2–64.7)
Yes	30.1 (26.8–33.3)	12.3 (11.1–13.5)	71.0 (69.1–72.8)	29.0 (27.2–30.9)	6.2 (5.0–7.5)	4.3 (3.6–4.9)	59.3 (54.9–63.7)	40.7 (36.3–45.1)
E-cigarette use								
No	64.2 (59.6–68.8)	27.7 (22.8–32.6)	69.9 (65.1–74.7)	30.1 (25.3–34.9)	23.9 (20.6–27.1)	28.9 (24.5–33.4)	45.2 (39.5–50.9)	54.8 (49.1–60.5)
Yes	30.3 (27.1–33.6)	12.5 (11.3–13.8)	70.8 (68.7–72.8)	29.2 (27.2–31.3)	6.4 (5.1–7.6)	4.7 (4.0–5.5)	57.4 (53.0–61.9)	42.6 (38.1–47.0)

Monitoring points in counties and county-level cities are defined as rural areas, while monitoring points in districts are defined as urban areas

*CI* confidence interval

**Table 4:** Types of alcoholic beverages consumed in the past year among adolescents in China, 2021

Variables	Beer		Wine		Spirit	
	Weighted % (95% CI)	OR (95% CI)	Weighted % (95% CI)	OR (95% CI)	Weighted % (95% CI)	OR (95% CI)
Total	71.1 (67.4–74.8)		69.4 (66.4–72.4)		17.5 (15.6–19.4)	
Gender						
Girls	61.8 (57.3–66.4)	1.0	76.6 (73.4–79.8)	1.0	11.3 (8.9–13.6)	1.0
Boys	77.5 (74.4–80.6)	2.1 (1.9–2.3)	64.2 (61.1–67.2)	0.5 (0.5–0.6)	21.9 (19.2–24.5)	2.2 (1.7–2.9)
Residence						
Urban	66.3 (63.4–69.2)	1.0	69.5 (65.9–73.2)	1.0	16.3 (14.6–18.1)	1.0
Rural	73.5 (68.2–78.8)	1.4 (1.0–1.9)	69.4 (65.2–73.5)	1.0 (0.8–1.3)	18.1 (15.3–20.8)	1.1 (0.9–1.4)
Region						
South	66.2 (62.7–69.6)	1.0	63.3 (56.6–70.0)	1.0	11.7 (9.4–13.9)	1.0
Northwest	69.0 (64.7–73.4)	1.1 (0.9–1.5)	69.3 (65.2–73.3)	1.3 (0.9–1.8)	22.4 (16.4–28.4)	2.2 (1.5–3.3)
Northeast	77.0 (74.0–80.0)	1.7 (1.4–2.2)	49.7 (42.9–56.4)	0.6 (0.4–0.9)	19.1 (14.1–24.2)	1.8 (1.2–2.7)
North	64.9 (59.3–70.6)	0.9 (0.7–1.3)	72.2 (66.5–77.8)	1.5 (1.0–2.3)	19.5 (18.4–20.7)	1.8 (1.5–2.3)
Central	68.5 (56.9–80.1)	1.1 (0.6–2.0)	70.3 (60.7–79.9)	1.4 (0.8–2.4)	16.9 (13.5–20.3)	1.5 (1.1–2.1)
East	75.8 (70.0–81.5)	1.6 (1.1–2.3)	75.6 (73.7–77.5)	1.8 (1.3–2.4)	17.5 (12.6–22.5)	1.6 (1.1–2.4)
Southwest	73.0 (67.5–78.4)	1.4 (1.0–1.9)	65.1 (59.0–71.2)	1.1 (0.7–1.6)	19.0 (13.7–24.3)	1.8 (1.2–2.7)
School type						
Middle school	65.2 (60.8–69.5)	1.0	71.9 (67.9–75.9)	1.0	14.8 (12.7–16.9)	1.0
High school	76.6 (73.0–80.1)	1.7 (1.5–2.0)	67.1 (63.9–70.2)	0.8 (0.7–1.0)	20.0 (16.0–24.0)	1.4 (1.0–2.0)
Academic high school	72.8 (69.0–76.6)	1.0	70.2 (66.9–73.4)	1.0	18.7 (14.3–23.2)	1.0
Vocational high school	84.6 (81.8–87.4)	2.1 (1.7–2.5)	59.9 (54.0–65.9)	0.6 (0.5–0.8)	22.9 (17.9–27.9)	1.3 (0.9–1.8)
Grade						
7 (12–< 14 y)	60.2 (52.1–68.3)	1.0	72.6 (67.2–78.0)	1.0	12.3 (9.1–15.5)	1.0
8 (13–< 15 y)	65.2 (60.0–70.4)	1.2 (1.0–1.6)	71.5 (67.8–75.3)	0.9 (0.8–1.2)	14.0 (11.8–16.2)	1.2 (0.9–1.5)
9 (14–< 16 y)	68.6 (64.2–72.9)	1.4 (1.0–2.0)	71.8 (66.8–76.8)	1.0 (0.8–1.2)	17.2 (13.2–21.2)	1.5 (1.0–2.2)
10 (15–< 17 y)	74.5 (68.0–81.1)	1.9 (1.6–2.3)	68.6 (64.0–73.3)	0.8 (0.6–1.1)	18.0 (11.2–24.9)	1.6 (0.8–2.9)
11 (16–< 18 y)	76.8 (74.0–79.6)	2.2 (1.6–3.1)	66.4 (63.1–69.7)	0.7 (0.6–1.0)	19.5 (15.1–23.8)	1.7 (1.3–2.2)
12 (17–< 19 y)	78.5 (74.8–82.3)	2.4 (1.9–3.1)	66.0 (62.7–69.3)	0.7 (0.6–0.9)	22.7 (19.1–26.4)	2.1 (1.6–2.8)

Monitoring points in counties and county-level cities are defined as rural areas, while monitoring points in districts are defined as urban areas

*CI* confidence interval, *OR* odds ratio

In past year drinkers, beer (71.1%) was the most popular alcoholic beverage consumed. Wine closely followed at 69.4% with grape wine specifically comprising 40.1%, fruit wine at 37.8% and rice wine at 32.3%. The consumption of spirits was 17.5%, with high-proof baijiu at 11.1% and low-proof baijiu at 12.5%. Boys exhibited a higher preference for beer (77.5%) and baijiu (21.9%) compared to girls (61.8%, 11.3%). There was no statistically significant difference in the preference for grape wine between sexes. Girls had a higher preference for fruit wine compared to boys (45.4% versus 32.3%). Drinking preferences did not differ significantly between urban and rural areas, except for a greater prevalence of yellow rice wine consumption in rural areas. Amongst beer drinkers, over half (51.9%) consume less than once per month, with the second most common frequency being 1–3 times monthly (34.2%). In the case of wine drinkers, 64.0% drink less than once a month, closely followed by those who drink 1–3 times a month (27.9%). For those who prefer high-proof baijiu, the most prevalent drinking frequency is also less than once a month (55.4%), with 1–3 times monthly next (30.1%). Similarly, low-proof baijiu drinkers mostly consume less than once a month (59.9%), with 1–3 times a month being the next most common frequency (29.2%). (Table 4, Supplementary Tables 8 and 9).

**Fig. 1:**
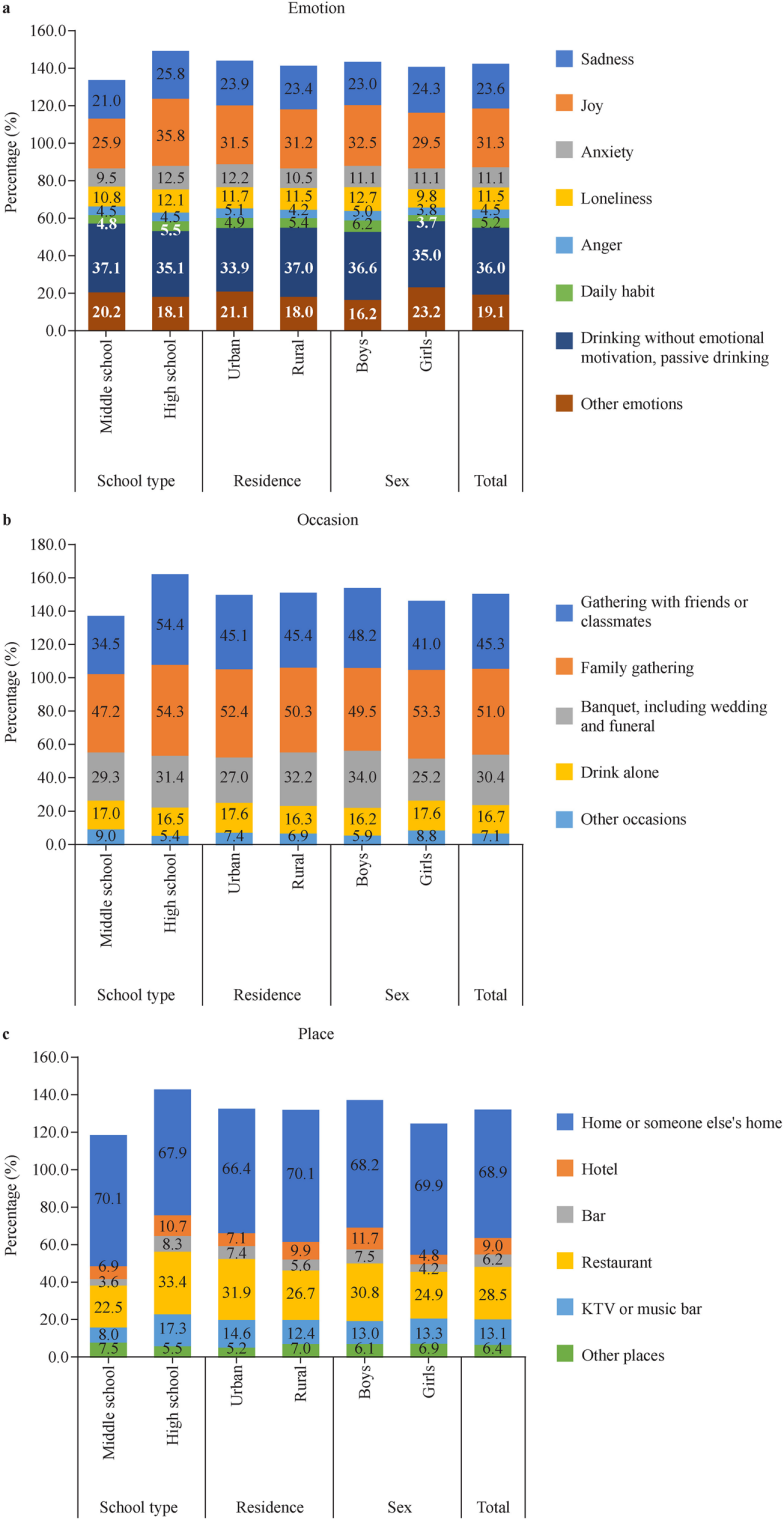
Typical emotions, occasions, and locations associated with alcohol use among middle and high school students in China, 2021. *KTV* karaoke television

The predominant cause of adolescent alcohol consumption is passive drinking without emotional motive (36.0%) followed by drinking during joy (31.3%) and sadness (23.6%); there was discernible disparities between sexes. The most common occasion for drinking is family gatherings (51.0%), followed by gatherings with friends or classmates (45.3%) and banquets (including weddings and funerals) (30.4%). Boys (48.2%) were more likely than girls (41.0%) to drink at gatherings with friends or classmates. The most common locations for drinking were either at one’s own or someone else’s home (68.9%), followed by restaurants (28.5%) and KTVs or music bars (13.1%). Boys were more likely than girls to drink in venues such as hotels (11.7% versus 4.8%) and bars (7.5% versus 4.2%). There is no urban–rural difference in the distribution of emotional motives, occasions and locations during drinking. (Fig. 1, Supplementary Table 10).

## Discussion

This nationwide survey aims to detail the prevalence and patterns of alcohol use among Chinese adolescents in middle and high school. There are three significant findings. Firstly, the study reveals that in 2021, the prevalence of alcohol use remained high, with lifetime, past year, and past month drinking percentages of 44. 1%, 32.7%, and 11.2% respectively. For comparison a United States based surveillance study of middle and high school students reported figures of 36.3%, 30.2%, 15.1% in 2021 [11]. Chinese students exhibited slightly higher percentages of lifetime and past year drinking and lower percentages for the past month. However, there was a higher prevalence of past-month alcohol use than seen in Korean (10.5% in 2020 and 2021) and Japan (9.5% in 2016) adolescents [12, 13]. A 2019 survey of about 100,000 European adolescents aged 15–16 across 35 countries revealed a lifetime drinking prevalence of 79% and a past-month consumption of 47% [14]. In contrast, Chinese adolescents demonstrate lower alcohol use, with 10th grade students (aged 15–< 17) reporting a lifetime prevalence of 52.2% and a past-month drinking incidence of 12.4%. This study demonstrates that Chinese adolescents display notably reduced incidences of drunkenness across all three assessment intervals, specifically lifetime, past year, and past month, with respective figures of 12.1%, 5.9%, and 1.6%. This is in contrast to their American peers who reported percentages of 21.1%, 15.5%, and 7.4% [11]. Moreover, among 15- and 16-year-olds students, drunkenness in the past month in China (1.8%) is below that in Europe at 13%.

Comparing data with the 2005 national survey [7], our study reveals a significant decrease in the prevalence of lifetime drinking among students in middle and high school grades 7–12, declining from 64.0% to 44.1%. In addition, the prevalence of past-month drinking also decreased, from 24.8% to 11.2%. Moreover, our analysis indicates a pronounced decrease in the prevalence of past-year drunkenness, decreasing from 14.6% in 2005 to the current 5.9%. This trend signifies a favorable transformation in the drinking patterns of Chinese adolescents, aligning with the widespread downward trend in adolescent alcohol consumption observed worldwide, particularly in regions like Europe, the United States, Canada and Japan [15–17]. The downward trend in alcohol consumption among Chinese teenagers could potentially be attributed to the enactment of two significant laws aimed at safeguarding the well-being of minors in China. Specifically, the Law on the Protection of Minors, enacted in 1992, emphasized the importance of preventing underage drinking and required parents and custodians to prevent and stop minors from excessive drinking [18, 19]. Subsequently, in 1999, the Law on the Prevention of Juvenile Delinquency was passed, advocating for prohibition of the sale of alcohol to minors [20, 21]. The most comprehensive effort to restrict underage drinking occurred in 2006 when, for the first time, the government mandated that the legal drinking age be set at age 18 years. Furthermore, since 2007, retailers have been required to visibly post signs indicating that they do not sell alcohol to minors, to request identification from customers whose age is unclear and to prevent alcohol consumption in places where minors congregate [18].

Secondly, a pronounced and consistent sex disparity is evident in alcohol use patterns. These differences are pervasive, encompassing key indicators such as the prevalence of drinking and drunkenness over a lifetime, past year and past month, frequency of drinking episodes, as well as early alcohol use and onset of drunkenness. Typically, boys exhibit a more severe trajectory in comparison to girls. In 2005, the sex ratio for lifetime drinking was 1.7 [7], compared to 1.6 in our study in 2021. And the sex ratio for past-month drinking was 1.9 in 2005 and also 1.9 in our study. These findings indicate that, despite the persisting disparities in drinking patterns between boys and girls over the past ten years, these differences are diminishing, matching the trend of decreasing sex differences among Chinese adults [4, 5], as well as among European and American adolescents [11, 14].

Thirdly, Chinese adolescents display disparities in alcohol consumption based on their urban-rural residence and geographical location. Rural adolescents encounter more severe drinking behavior compared to their urban peers. Although the study did not reveal a statistically significant difference in the prevalence of lifetime drinking between urban and rural areas (*P* = 0.06), it showed that the prevalence of drinking in the past year and past month in rural areas was 1.3 times higher than in urban areas. Furthermore, whether viewed over a lifetime, the past year or the past month, the incidence of drunkenness in rural areas is roughly 1.6 times greater than in urban areas. Notably, this trend contrasts with the pattern observed among Chinese adults [5, 22]. Easier access to alcohol for rural adolescents, coupled with less parental supervision, especially with millions of children “left-behind” whose parents work in cities, may explain their heavier alcohol consumption compared to urban teens [9].

This study revealed a similar trend in the prevalence of drinking and drunkenness, indicating that regions with elevated alcohol drinking also experienced higher incidences of drunkenness. In particular, Central China ranks highest in terms of both alcohol drinking and drunkenness, as well as the early onset of these behaviors. This is presumably due to the deeply rooted drinking culture in this region, which fosters widespread acceptance. Conversely, the lowest prevalence of drinking across all time frames and the lowest lifetime and past-year prevalence of drunkenness was reported in Northwest China. This may be attributed to the distinct cultural background of the region as a predominantly ethnic minority community; these ethnic groups have stricter attitudes towards alcohol consumption.

The key strength of this study lies in its large, nationally representative sample of middle and high school students, coupled with a remarkably high participation rate. This study estimated the prevalence and related patterns of alcohol use, serving as a baseline for future research and intervention. A limitation was that behavioral data were self-reported and might be subject to recall bias. The questionnaire design drew inspiration from relevant monitoring guidelines for alcohol consumption of the World Health Organization [23], as well as surveys on adolescent alcohol consumption conducted in the United States and Europe; this ensures measurement accuracy. In addition, the large sample size of this study helps mitigate for potential biases.

In conclusion, this study details a concerning prevalence of alcohol use among Chinese adolescents; this is despite a downward trend. Moreover, there was roughly one-third reporting past-year drinking. Boys drink more heavily than girls and significant geographical disparities exist. This study reports that adolescent drinking is mainly passive during social gatherings with family or peers and within private homes. This highlights a vital window to address and reduce underage drinking. In developing strategies to curb adolescent drinking, special attention must be devoted to the influential roles of both parents and peer groups. Low alcohol use among ethnic minorities in Northwest China highlights the effect of drinking culture on adolescents and underscores the importance of curbing permissive social norms and attitudes. Despite enacting laws to reduce underage drinking [19, 21], China faces enforcement gaps. The WHO’s recommendations and global anti-adolescent drinking efforts offer China crucial insights [24, 25].

## Supplementary Information

Below is the link to the electronic supplementary material.

Supplementary file1 (DOCX 212 KB)

## Data Availability

The data of this study are available on reasonable request to the corresponding author.
