# Supplementary material for: Alcohol consumption is prevalent among Chinese adolescents: a national survey
Source: World J Pediatr. 2025 Dec 26;22(1):142–54. doi: 10.1007/s12519-025-00994-4 (PMC12894188; doi:10.1007/s12519-025-00994-4)
Supplement: Supplementary file 1 — Supplementary file1 (DOCX 212 KB) [file 12519_2025_994_MOESM1_ESM.docx]

**Supplementary appendix**

**Table S1. Characteristics of middle and high school students in China, 2021**

| **Characteristics** | **Middle school students** | |  | **High school students** | | | | |
| --- | --- | --- | --- | --- | --- | --- | --- | --- |
|  |  |  |  | **Academic high school** | |  | **Vocational high school** | |
|  | **Unweighted,  No.** | **Weighted %  (95%CI)** |  | **Unweighted,  No.** | **Weighted %  (95%CI)** |  | **Unweighted,  No.** | **Weighted %  (95%CI)** |
| Total | 29694 |  |  | 20586 |  |  | 7056 |  |
| Sex |  |  |  |  |  |  |  |  |
| Boys | 15481 | 53.5(52.6-54.5) |  | 10174 | 49.0(46.0-52.0) |  | 3974 | 56.1(47.3-64.9) |
| Girls | 14213 | 46.5(45.5-47.4) |  | 10412 | 51.0(48.0-54.0) |  | 3082 | 43.9(35.1-52.7) |
| Residence |  |  |  |  |  |  |  |  |
| Urban | 17538 | 37.2(31.2-43.2) |  | 12390 | 39.6(34.1-45.1) |  | 4145 | 32.7(23.4-42.0) |
| Rural | 12156 | 62.8(56.8-68.8) |  | 8196 | 60.4(54.9-65.9) |  | 2911 | 67.3(58.0-76.6) |
| Region |  |  |  |  |  |  |  |  |
| Northeast | 3105 | 4.7(3.4-6.0) |  | 2307 | 6.5(3.3-9.7) |  | 426 | 4.1(3.1-5.2) |
| Northwest | 3122 | 7.6(5.7-9.4) |  | 2271 | 7.6(6.1-9.2) |  | 766 | 7.8(5.3-10.3) |
| North | 4057 | 11.5(7.7-15.3) |  | 2613 | 11.8(6.7-16.9) |  | 1286 | 5.9(2.7-9.0) |
| Central | 5458 | 19.5(16.1-22.9) |  | 3460 | 18.5(13.9-23.2) |  | 1522 | 16.9(11.7-22.1) |
| East | 4488 | 29.0(19.6-38.4) |  | 3625 | 28.0(23.7-32.3) |  | 829 | 33.4(21.6-45.3) |
| South | 4245 | 11.8(9.3-14.3) |  | 3219 | 11.1(8.2-14.1) |  | 941 | 10.8(1.6-20.0) |
| Southwest | 5219 | 16.0(11.8-20.1) |  | 3091 | 16.4(13.5-19.3) |  | 1286 | 21.1(13.9-28.2) |
| Grade |  |  |  |  |  |  |  |  |
| 7 (12–< 14 y) | 10244 | 34.2(33.1-35.4) |  | – | – |  | – | – |
| 8 (13–< 15 y) | 9823 | 33.5(32.7-34.4) |  | – | – |  | – | – |
| 9 (14–< 16 y) | 9627 | 32.2(31.2-33.3) |  | – | – |  | – | – |
| 10 (15–< 17 y) | – | – |  | 6770 | 35.8(33.8-37.8) |  | 2605 | 38.8(29.5-48.0) |
| 11 (16–< 18 y) | – | – |  | 7007 | 32.3(31.2-33.4) |  | 2414 | 32.1(26.7-37.6) |
| 12 (17–< 19 y) | – | – |  | 6809 | 31.9(30.3-33.4) |  | 2037 | 29.1(15.2-43.0) |

**Table S2. Prevalence of alcohol drinking over a lifetime, past year, and past month among middle school students in China, 2021**

|  | **Lifetime drinking** | | | |  | **Past year drinking** | | | | | **Past month drinking** | | | | |  |
| --- | --- | --- | --- | --- | --- | --- | --- | --- | --- | --- | --- | --- | --- | --- | --- | --- |
|  | **Unweighted, No.** | **Weighted %  (95% CI)** | **OR (95%CI)** |  | | **Unweighted, No.** | **Weighted %  (95% CI)** | **OR (95%CI)** | |  | | **Unweighted, No.** | | **Weighted % (95% CI)** | **OR (95%CI)** |  |
| **Total** | 9718 | 35.8(31.1-40.6) |  |  | | 7260 | 26.4(22.6-30.3) | |  | | |  | 2302 | 8.9(7.6-10.1) |  |  |
| Gender |  |  |  |  | |  |  | |  | | |  |  |  |  |  |
| Girls | 4078 | 30.8(26.4-35.2) | 1.0 |  | | 2961 | 22.3(19.0-25.6) | | 1.0 | | |  | 855 | 6.4(4.8-7.9) | 1.0 |  |
| Boys | 5640 | 40.3(35.1-45.4) | 1.5(1.4-1.6) |  | | 4299 | 30.0(25.7-34.4) | | 1.5(1.4-1.6) | | |  | 1447 | 11.0(9.7-12.3) | 1.8(1.5-2.3) |  |
| Residence |  |  |  |  | |  |  | |  | | |  |  |  |  |  |
| Urban | 5493 | 32.2(30.1-34.4) | 1.0 |  | | 3926 | 22.7(21.1-24.3) | | 1.0 | | |  | 1205 | 7.0(6.4-7.6) | 1.0 |  |
| Rural | 4225 | 38.0(30.1-45.9) | 1.3(0.9-1.8) |  | | 3334 | 28.6(22.2-35.0) | | 1.4(1.0-1.9) | | |  | 1097 | 10.0(7.9-12) | 1.5(1.1-1.9) |  |
| Region |  |  |  |  | |  |  | |  | | |  |  |  |  |  |
| Northwest | 776 | 25.5(17.9-33.1) | 1.0 |  | | 578 | 18.8(13.1-24.5) | | 1.0 | | |  | 198 | 6.4(4.5-8.4) | 1.0 |  |
| Northeast | 670 | 22.6(15.3-29.9) | 0.9(0.5-1.5) |  | | 516 | 17.3(9.8-24.8) | | 0.9(0.5-1.7) | | |  | 184 | 6.3(3.8-8.8) | 1.0(0.6-1.7) |  |
| North | 1327 | 33.6(31.0-36.3) | 1.5(1.0-2.2) |  | | 911 | 22.7(20.9-24.6) | | 1.3(0.9-1.9) | | |  | 290 | 7.2(6.4-8.0) | 1.1(0.8-1.6) |  |
| Central | 2173 | 43.1(35.7-50.4) | 2.2(1.3-3.6) |  | | 1766 | 35.0(26.3-43.6) | | 2.3(1.4-4.0) | | |  | 565 | 11.3(7.9-14.7) | 1.8(1.2-2.9) |  |
| East | 1483 | 37.3(21.9-52.6) | 1.7(0.8-3.7) |  | | 1045 | 25.8(15.0-36.7) | | 1.5(0.8-3.0) | | |  | 347 | 9.8(6.1-13.5) | 1.6(0.9-2.7) |  |
| South | 1418 | 32.7(27.3-38.1) | 1.4(0.9-2.3) |  | | 1106 | 25.4(21.7-29.1) | | 1.5(1.0-2.2) | | |  | 344 | 7.9(6.3-9.5) | 1.2(0.8-1.8) |  |
| Southwest | 1871 | 37.3(31.2-43.4) | 1.7(1.1-2.8) |  | | 1338 | 26.9(21.7-32.2) | | 1.6(1.0-2.5) | | |  | 374 | 8.0(6.2-9.8) | 1.3(0.8-1.9) |  |
| Grade |  |  |  |  | |  |  | |  | | |  |  |  |  |  |
| 7(12–< 14 y) | 2701 | 29.8(24.5-35.1) | 1.0 |  | | 1955 | 21.6(17.7-25.4) | | 1.0 | | |  | 554 | 6.9(6.1-7.6) | 1.0 |  |
| 8(13–< 15 y) | 3244 | 34.8(29.4-40.3) | 1.3(1.1-1.5) |  | | 2387 | 24.4(19.0-29.9) | | 1.2(1.0-1.4) | | |  | 782 | 8.3(6.1-10.5) | 1.2(0.9-1.7) |  |
| 9(14–< 16 y) | 3773 | 43.3(38.6-48.1) | 1.8(1.6-2.1) |  | | 2918 | 33.7(30.4-37.0) | | 1.9(1.6-2.1) | | |  | 966 | 11.6(9.5-13.8) | 1.8(1.4-2.3) |  |
| Cigarette use |  |  |  |  | |  |  | |  | | |  |  |  |  |  |
| No | 9049 | 34.4(29.9-38.9) | 1.0 |  | | 6650 | 25.0(21.5-28.5) | | 1.0 | | |  | 1992 | 8.0(6.9-9) | 1.0 |  |
| Yes | 662 | 88.1(84.3-91.9) | 14.1(9.7-20.5) |  | | 605 | 78.0(73.8-82.2) | | 10.6(8.3-13.6) | | |  | 308 | 41.4(37.1-45.8) | 8.2(7.0-9.5) |  |
| E-Cigarette use |  |  |  |  | |  |  | |  | | |  |  |  |  |  |
| No | 9072 | 34.6(30.1-39.2) | 1.0 |  | | 6681 | 25.3(21.7-28.9) | | 1.0 | | |  | 1998 | 8.1(7.0-9.2) | 1.0 |  |
| Yes | 578 | 87.7(79.7-95.6) | 13.4(7.6-23.8) |  | | 526 | 77.6(69.9-85.3) | | 10.2(7.6-13.9) | | |  | 284 | 43.8(37.0-50.7) | 8.9(7.1-11.1) |  |

Abbreviation: CI, confidence interval. Monitoring points in counties and county-level cities are defined as rural areas, while monitoring points in districts are defined as urban areas.

| **Table S3: Prevalence of alcohol drinking over a lifetime, past year, and past month among high school students in China, 2021** | | | | | | | | | | | | |
| --- | --- | --- | --- | --- | --- | --- | --- | --- | --- | --- | --- | --- |
|  | **Lifetime drinking** | | |  | **Past year drinking** | | | **Past month drinking** | | | |  |
|  | **Unweighted, No.** | **Weighted %  (95% CI)** | **OR (95%CI)** |  | **Unweighted, No.** | **Weighted %  (95% CI)** | **OR (95%CI)** |  | **Unweighted, No.** | **Weighted % (95% CI)** | **OR (95%CI)** |  |
| Total | 14367 | 55.1(51.9-58.3) |  |  | 10870 | 41.0(38.7-43.4) |  |  | 3930 | 14.4(12.8-16.0) |  |  |
| Gender |  |  |  |  |  |  |  |  |  |  |  |  |
| Girls | 8292 | 47.2(43.0-51.3) | 1.0 |  | 4202 | 31.9(29.6-34.3) | 1.0 |  | 1352 | 9.9(8.6-11.3) | 1.0 |  |
| Boys | 6075 | 62.7(59.9-65.6) | 1.9(1.7-2.1) |  | 6668 | 49.8(47.0-52.6) | 2.1(1.9-2.3) |  | 2578 | 18.7(16.5-20.9) | 2.1(1.8-2.4) |  |
| Residence |  |  |  |  |  |  |  |  |  |  |  |  |
| Urban | 8356 | 50.2(47.6-52.8) | 1.0 |  | 6116 | 36.7(34.6-38.8) | 1.0 |  | 2254 | 13.5(12.4-14.7) | 1.0 |  |
| Rural | 6011 | 58.0(53.4-62.7) | 1.4(1.1-1.7) |  | 4754 | 43.6(40.3-47.0) | 1.3(1.1-1.6) |  | 1676 | 14.9(12.5-17.4) | 1.1(0.9-1.4) |  |
| Region |  |  |  |  |  |  |  |  |  |  |  |  |
| Northwest | 1318 | 44.5(31.9-57.2) | 1.0 |  | 1028 | 34.1(24.6-43.7) | 1.0 |  | 405 | 13.5(9.5-17.6) | 1.0 |  |
| Northeast | 1229 | 46.0(41.1-50.9) | 1.1(0.6-1.8) |  | 962 | 34.7(31.1-38.2) | 1.0(0.6-1.6) |  | 389 | 13.8(12.9-14.7) | 1.0(0.7-1.5) |  |
| North | 1917 | 47.6(44.9-50.2) | 1.1(0.7-1.9) |  | 1473 | 35.3(32.5-38.0) | 1.1(0.7-1.6) |  | 608 | 13.3(10.8-15.8) | 1.0(0.7-1.5) |  |
| Central | 2893 | 62.5(52.4-72.5) | 2.1(1.1-4.0) |  | 2237 | 47.2(39.3-55.1) | 1.7(1.0-2.9) |  | 737 | 15.1(11.3-19.0) | 1.1(0.7-1.8) |  |
| East | 2418 | 57.6(52.2-63.0) | 1.7(1.0-3.0) |  | 1670 | 40.3(36.7-43.9) | 1.3(0.8-2.0) |  | 551 | 13.8(9.8-17.9) | 1.0(0.6-1.7) |  |
| South | 2171 | 51.6(43.9-59.3) | 1.3(0.7-2.4) |  | 1662 | 40.0(35.7-44.3) | 1.3(0.8-2.0) |  | 590 | 14.6(11.1-18.1) | 1.1(0.7-1.7) |  |
| Southwest | 2421 | 57.3(50.7-64.0) | 1.7(0.9-3.0) |  | 1838 | 44.9(39.1-50.8) | 1.6(1.0-2.6) |  | 650 | 15.7(12.5-19.0) | 1.2(0.8-1.8) |  |
| Grade |  |  |  |  |  |  |  |  |  |  |  |  |
| 10 (15–< 17 y) | 4470 | 52.2(47.6-56.7) | 1.0 |  | 3430 | 39.4(36.4-42.4) | 1.0 |  | 1145 | 12.4(10.8-14.0) | 1.0 |  |
| 11 (16–< 18 y) | 4889 | 54.7(51.2-58.2) | 1.1(0.9-1.4) |  | 3632 | 39.7(35.6-43.8) | 1.0(0.9-1.2) |  | 1335 | 14.8(13.3-16.3) | 1.2(1.0-1.4) |  |
| 12 (17–< 19 y) | 5008 | 59.0(54.0-64.0) | 1.3(1.1-1.6) |  | 3808 | 44.5(40.0-48.9) | 1.2(1.0-1.5) |  | 1450 | 16.4(13.4-19.5) | 1.4(1.2-1.6) |  |
| Cigarette use |  |  |  |  |  |  |  |  |  |  |  |  |
| No | 13101 | 53.1(50.0-56.2) | 1.0 |  | 9689 | 38.7(36.5-40.8) | 1.0 |  | 3217 | 12.5(11.0-14.0) | 1.0 |  |
| Yes | 1259 | 93.7(91.9-95.5) | 13.2(10.0-17.5) |  | 1174 | 85.9(82.6-89.1) | 9.6(7.5-12.3) |  | 710 | 51.0(45.6-56.3) | 7.3(5.9-9.0) |  |
| E-Cigarette use |  |  |  |  |  |  |  |  |  |  |  |  |
| No | 13434 | 53.8(50.6-57.0) | 1.0 |  | 9998 | 39.6(37.3-41.8) | 1.0 |  | 3425 | 13.3(11.6-14.9) | 1.0 |  |
| Yes | 861 | 95.8(94.1-97.4) | 19.4(13.3-28.2) |  | 812 | 87.9(84.6-91.3) | 11.1(8.2-15.0) |  | 479 | 50.4(44.7-56.1) | 6.6(5.2-8.5) |  |
| *Academic high school* |  |  |  |  |  |  |  |  |  |  |  |  |
| Total | 10533 | 54.1(50.6-57.6) |  |  | 7782 | 39.4(36.6-42.2) |  |  | 2630 | 12.8(11.4-14.2) |  |  |
| Gender |  |  |  |  |  |  |  |  |  |  |  |  |
| Girls | 4674 | 47.5(42.8-52.1) | 1.0 |  | 3142 | 31.3(28.4-34.2) | 1.0 |  | 936 | 9.0(8.0-10.0) | 1.0 |  |
| Boys | 5859 | 61.1(57.5-64.8) | 1.7(1.4-2.1) |  | 4640 | 47.8(44.2-51.4) | 2.0(1.7-2.3) |  | 1694 | 16.8(14.6-18.9) | 2.0(1.9-2.3) |  |
| Residence |  |  |  |  |  |  |  |  |  |  |  |  |
| Urban | 6162 | 49.0(46.2-51.8) | 1.0 |  | 4395 | 34.8(32.7-36.8) | 1.0 |  | 1499 | 12.0(10.9-13.0) | 1.0 |  |
| Rural | 4371 | 57.5(52.2-62.7) | 1.4(1.1-1.8) |  | 3387 | 42.4(38.1-46.7) | 1.4(1.1-1.7) |  | 1131 | 13.3(11.1-15.5) | 1.1(0.9-1.4) |  |
| Region |  |  |  |  |  |  |  |  |  |  |  |  |
| Northwest | 945 | 43.3(30.7-56.0) | 1.0 |  | 727 | 32.9(23.7-42.1) | 1.0 |  | 281 | 12.9(9.1-16.6) | 1.0 |  |
| Northeast | 1003 | 45.3(40.1-50.6) | 1.1(0.6-1.9) |  | 782 | 34.0(30.9-37.0) | 1.1(0.7-1.6) |  | 314 | 13.6(12.6-14.5) | 1.1(0.8-1.5) |  |
| North | 1277 | 48.1(46.2-50.0) | 1.2(0.7-2.0) |  | 964 | 35.3(32.8-37.8) | 1.1(0.7-1.7) |  | 366 | 12.8(10.1-15.6) | 1.0(0.7-1.5) |  |
| Central | 2010 | 62.8(49.8-75.9) | 2.2(1.0-4.7) |  | 1484 | 45.7(34.9-56.6) | 1.7(0.9-3.1) |  | 419 | 12.6(8.1-17.2) | 1.0(0.6-1.7) |  |
| East | 1966 | 57.4(54.1-60.8) | 1.8(1.0-3.0) |  | 1336 | 39.3(35.4-43.3) | 1.3(0.8-2.1) |  | 428 | 12.2(10.2-14.3) | 0.9(0.6-1.4) |  |
| South | 1671 | 50.5(43.2-57.7) | 1.3(0.7-2.4) |  | 1261 | 38.2(33.3-43.1) | 1.3(0.8-2.0) |  | 397 | 11.8(9.6-14.0) | 0.9(0.6-1.3) |  |
| Southwest | 1661 | 53.9(45.8-62.0) | 1.5(0.8-2.8) |  | 1228 | 41.1(34.7-47.6) | 1.4(0.9-2.3) |  | 425 | 14.2(9.1-19.3) | 1.1(0.7-1.9) |  |
| Grade |  |  |  |  |  |  |  |  |  |  |  |  |
| 10 (15–< 17 y) | 3106 | 49.2(45.3-53.1) | 1.0 |  | 2345 | 36.0(32.0-40.1) | 1.0 |  | 735 | 10.0(8.3-11.8) | 1.0 |  |
| 11 (16–< 18 y) | 3627 | 54.3(49.9-58.7) | 1.2(1.1-1.4) |  | 2636 | 38.8(35.0-42.7) | 1.1(1.0-1.3) |  | 931 | 13.9(12.0-15.8) | 1.4(1.1-1.8) |  |
| 12 (17–< 19 y) | 3800 | 59.5(54.7-64.3) | 1.5(1.2-1.9) |  | 2801 | 43.7(39.5-47.9) | 1.4(1.1-1.8) |  | 964 | 14.8(11.7-17.9) | 1.6(1.1-2.2) |  |
| Cigarette use |  |  |  |  |  |  |  |  |  |  |  |  |
| No | 9901 | 52.7(49.4-56.1) | 1.0 |  | 7191 | 37.7(35.0-40.4) | 1.0 |  | 2263 | 11.3(10.2-12.5) | 1.0 |  |
| Yes | 627 | 93.4(90.7-96.2) | 12.8(8.3-19.6) |  | 586 | 86.2(81.6-90.7) | 10.3(7.0-15.1) |  | 366 | 53.6(43.5-63.6) | 9.0(6.2-13.0) |  |
| E-Cigarette use |  |  |  |  |  |  |  |  |  |  |  |  |
| No | 10062 | 53.2(49.8-56.6) | 1.0 |  | 7335 | 38.3(35.5-41.0) | 1.0 |  | 2385 | 12.0(10.6-13.3) | 1.0 |  |
| Yes | 429 | 96.0(93.9-98.1) | 21.0(12.9-34.3) |  | 410 | 88.6(84.7-92.4) | 12.5(8.6-18.1) |  | 231 | 48.6(40.7-56.4) | 6.9(5.2-9.2) |  |
| *Vocational high school* |  |  |  |  |  |  |  |  |  |  |  |  |
| Total | 3834 | 57.4(51.0-63.8) |  |  | 3088 | 45.0(41.0-49.0) |  |  | 1300 | 18.2(15.0-21.5) |  |  |
| Gender |  |  |  |  |  |  |  |  |  |  |  |  |
| Girls | 1401 | 46.4(40.8-51.9) | 1.0 |  | 1060 | 33.7(30.2-37.1) | 1.0 |  | 416 | 12.5(8.9-16.1) | 1.0 |  |
| Boys | 2433 | 66.0(59.8-72.2) | 2.2(1.8-2.8) |  | 2028 | 53.8(49.5-58.2) | 2.3(1.8-2.9) |  | 884 | 22.7(19.7-25.8) | 2.1(1.5-2.8) |  |
| Residence |  |  |  |  |  |  |  |  |  |  |  |  |
| Urban | 2194 | 53.7(49.1-58.4) | 1.0 |  | 1721 | 42.3(38.1-46.5) | 1.0 |  | 755 | 17.9(15.1-20.7) | 1.0 |  |
| Rural | 1640 | 59.2(50.1-68.3) | 1.2(0.8-1.9) |  | 1367 | 46.3(40.9-51.6) | 1.2(0.9-1.5) |  | 545 | 18.4(13.8-23.0) | 1.0(0.7-1.5) |  |
| Region |  |  |  |  |  |  |  |  |  |  |  |  |
| North | 640 | 44.8(33.3-56.4) | 1.0 |  | 509 | 35.2(24.8-45.6) | 1.0 |  | 242 | 15.6(10.7-20.4) | 1.0 |  |
| Northwest | 373 | 47.4(34.9-59.9) | 1.1(0.6-2.2) |  | 301 | 37.0(26.5-47.4) | 1.1(0.6-2.0) |  | 124 | 15.1(9.8-20.4) | 1.0(0.6-1.7) |  |
| Northeast | 226 | 48.6(45.4-51.8) | 1.2(0.7-1.9) |  | 180 | 37.1(35.9-38.3) | 1.1(0.7-1.7) |  | 75 | 14.5(13.9-15.1) | 0.9(0.6-1.3) |  |
| Central | 883 | 61.5(54.8-68.1) | 2.0(1.1-3.4) |  | 753 | 51.0(43.6-58.3) | 1.9(1.1-3.3) |  | 318 | 21.7(15.3-28.1) | 1.5(0.9-2.5) |  |
| East | 452 | 58.0(41.2-74.7) | 1.7(0.7-3.9) |  | 334 | 42.3(33.6-51.0) | 1.4(0.8-2.4) |  | 123 | 17.0(8.4-25.5) | 1.1(0.5-2.3) |  |
| South | 500 | 54.2(42.1-66.2) | 1.5(0.7-2.9) |  | 401 | 44.5(37.6-51.3) | 1.5(0.9-2.5) |  | 193 | 21.3(12.4-30.1) | 1.5(0.8-2.8) |  |
| Southwest | 760 | 63.7(56.2-71.1) | 2.2(1.2-3.8) |  | 610 | 51.9(43.3-60.5) | 2.0(1.1-3.5) |  | 225 | 18.6(16.5-20.7) | 1.2(0.8-1.8) |  |
| Grade |  |  |  |  |  |  |  |  |  |  |  |  |
| 10 (15–< 17 y) | 1364 | 58.6(47.0-70.2) | 1.0 |  | 1085 | 46.6(38.5-54.8) | 1.0 |  | 410 | 17.5(12.6-22.4) | 1.0 |  |
| 11 (16–< 18 y) | 1262 | 55.7(50.7-60.7) | 0.9(0.6-1.4) |  | 996 | 41.7(34.7-48.6) | 0.8(0.5-1.4) |  | 404 | 16.9(14.0-19.8) | 1.0(0.7-1.4) |  |
| 12 (17–< 19 y) | 1208 | 57.6(49.4-65.7) | 1.0(0.6-1.5) |  | 1007 | 46.4(37.8-55.0) | 1.0(0.7-1.4) |  | 486 | 20.8(14.1-27.5) | 1.2(0.9-1.8) |  |
| Cigarette use |  |  |  |  |  |  |  |  |  |  |  |  |
| No | 3200 | 53.9(47.3-60.6) | 1.0 |  | 2498 | 41.1(37.5-44.8) | 1.0 |  | 954 | 15.4(11.7-19.0) | 1.0 |  |
| Yes | 632 | 94.0(92.0-96.1) | 13.5(9.0-20.1) |  | 588 | 85.7(81.7-89.6) | 8.5(6.4-11.4) |  | 344 | 48.5(42.6-54.4) | 5.2(3.4-8.0) |  |
| E-Cigarette use |  |  |  |  |  |  |  |  |  |  |  |  |
| No | 3372 | 55.2(48.5-61.9) | 1.0 |  | 2663 | 42.7(38.8-46.6) | 1.0 |  | 1040 | 16.4(12.9-19.9) | 1.0 |  |
| Yes | 432 | 95.5(93.0-98.1) | 17.3(9.4-31.9) |  | 402 | 87.3(82.7-92.0) | 9.2(6.2-13.9) |  | 248 | 52.2(46.0-58.5) | 5.6(3.7-8.5) |  |

Abbreviation: CI, confidence interval.

Monitoring points in counties and county-level cities are defined as rural areas, while monitoring points in districts are defined as urban areas.

**Table S4: Prevalence of alcohol drunkenness over a lifetime, past year, and past month among middle school students in China, 2021**

|  | **Lifetime drunkenness** | | |  | **Past year drunkenness** | | |  | **Past month drunkenness** | | |
| --- | --- | --- | --- | --- | --- | --- | --- | --- | --- | --- | --- |
|  | **Unweighted,  No.** | **Weighted %  (95% CI)** | **OR (95%CI)** |  | **Unweighted,  No.** | **Weighted %  (95% CI)** | **OR (95%CI)** |  | **Unweighted,  No.** | **Weighted %  (95% CI)** | **OR (95%CI)** |
| Total | 2415 | 9.2(7.2-11.1) |  |  | 1130 | 4.2(3.2-5.1) |  |  | 329 | 1.3(1.0-1.7) |  |
| Gender |  |  |  |  |  |  |  |  |  |  |  |
| Girls | 871 | 6.6(5.0-8.3) | 1.0 |  | 401 | 2.9(2.1-3.8) | 1.0 |  | 111 | 0.9(0.5-1.2) | 1.0 |
| Boys | 1544 | 11.8(9.3-14.3) | 1.9(1.6-2.2) |  | 729 | 5.2(4.1-6.4) | 1.8(1.5-2.3) |  | 218 | 1.7(1.3-2.2) | 2.0(1.5-2.8) |
| Residence |  |  |  |  |  |  |  |  |  |  |  |
| Urban | 1154 | 6.6(5.5-7.7) | 1.0 |  | 523 | 2.9(2.4-3.5) | 1.0 |  | 142 | 0.8(0.6-1.1) | 1.0 |
| Rural | 1261 | 11.1(7.7-14.4) | 1.8(1.2-2.6) |  | 607 | 4.9(3.3-6.5) | 1.7(1.2-2.5) |  | 187 | 1.6(1.0-2.2) | 2.0(1.2-3.2) |
| Region |  |  |  |  |  |  |  |  |  |  |  |
| North | 203 | 4.6(3.5-5.7) | 1.0 |  | 92 | 2.1(1.3-2.8) | 1.0 |  | 27 | 0.6(0.3-1.0) | 1.0 |
| Northeast | 214 | 7.1(3.5-10.6) | 1.6(0.9-2.9) |  | 121 | 3.9(2.3-5.5) | 1.9(1.1-3.4) |  | 28 | 1.0(0.7-1.2) | 1.5(0.8-2.9) |
| Northwest | 209 | 7.0(4.0-10.0) | 1.6(0.9-2.6) |  | 110 | 3.6(2.2-4.9) | 1.8(1.0-3.1) |  | 33 | 1.0(0.3-1.7) | 1.6(0.6-3.9) |
| Central | 607 | 13.1(10.0-16.3) | 3.2(2.2-4.6) |  | 284 | 6.0(3.7-8.4) | 3.1(1.8-5.4) |  | 101 | 2.3(0.9-3.6) | 3.6(1.5-8.5) |
| East | 317 | 8.8(3.8-13.8) | 2.0(1.0-3.9) |  | 137 | 3.7(1.9-5.5) | 1.8(1.0-3.4) |  | 38 | 1.1(0.6-1.6) | 1.8(0.9-3.7) |
| South | 325 | 7.5(5.2-9.8) | 1.7(1.1-2.6) |  | 142 | 3.4(2.4-4.3) | 1.7(1.0-2.7) |  | 43 | 1.1(0.8-1.3) | 1.7(0.9-3.2) |
| Southwest | 540 | 12.5(6.6-18.4) | 3.0(1.6-5.3) |  | 244 | 5.4(2.0-8.7) | 2.7(1.3-5.8) |  | 59 | 1.4(0.4-2.5) | 2.3(0.9-5.9) |
| Grade |  |  |  |  |  |  |  |  |  |  |  |
| 7(12–< 14 y) | 659 | 8.0(6.6-9.5) | 1.0 |  | 297 | 3.5(2.7-4.3) | 1.0 |  | 79 | 1.1(0.6-1.6) | 1.0 |
| 8(13–< 15 y) | 760 | 8.3(6.0-10.7) | 1.0(0.8-1.3) |  | 367 | 3.8(2.5-5.1) | 1.1(0.7-1.6) |  | 112 | 1.4(0.7-2.1) | 1.4(0.6-3.2) |
| 9(14–< 16 y) | 996 | 11.9(9.0-14.8) | 1.5(1.2-1.9) |  | 466 | 5.3(3.9-6.7) | 1.6(1.2-2.0) |  | 138 | 1.5(0.8-2.2) | 1.4(0.7-3.0) |
| Cigarette use |  |  |  |  |  |  |  |  |  |  |  |
| No | 2047 | 8.2(6.6-9.8) | 1.0 |  | 859 | 3.3(2.7-3.9) | 1.0 |  | 214 | 0.9(0.7-1.1) | 1.0 |
| Yes | 364 | 53.3(45.0-61.6) | 12.7(9.2-17.5) |  | 267 | 36.4(31.0-41.8) | 16.9(13.7-20.9) |  | 113 | 15.9(12.3-19.5) | 20.5(15.2-27.7) |
| E-Cigarette use |  |  |  |  |  |  |  |  |  |  |  |
| No | 2087 | 8.4(6.7-10.2) | 1.0 |  |  |  | 1.0 |  |  |  | 1.0 |
| Yes | 288 | 49.4(41.9-56.8) | 10.6(8.8-12.6) |  | 201 | 32.9(27.7-38.1) | 13.7(11.3-16.6) |  | 82 | 13.2(8.8-17.6) | 15.1(10.8-21.2) |

Abbreviation: CI, confidence interval.

Monitoring points in counties and county-level cities are defined as rural areas, while monitoring points in districts are defined as urban areas.

**Table S5: Prevalence of alcohol drunkenness over a lifetime, past year, and past month among high school students in China, 2021**

|  | **Lifetime drunkenness** | | |  | **Past year drunkenness** | | |  | **Past month drunkenness** | | |
| --- | --- | --- | --- | --- | --- | --- | --- | --- | --- | --- | --- |
|  | **Unweighted,  No.** | **Weighted %  (95% CI)** | **OR (95%CI)** |  | **Unweighted,  No.** | **Weighted %  (95% CI)** | **OR (95%CI)** |  | **Unweighted,  No.** | **Weighted %  (95% CI)** | **OR (95%CI)** |
| Total | 3810 | 15.4(13.7-17.2) |  |  | 2041 | 8.1(6.8-9.3) |  |  | 549 | 1.9(1.6-2.3) |  |
| Gender |  |  |  |  |  |  |  |  |  |  |  |
| Girls | 1259 | 11.2(8.3-14.1) | 1.0 |  | 571 | 4.8(3.3-6.4) | 1.0 |  | 154 | 1.1(0.8-1.4) | 1.0 |
| Boys | 2551 | 20.4(18.5-22.3) | 2.0(1.5-2.8) |  | 1470 | 11.2(9.8-12.6) | 2.5(1.8-3.4) |  | 395 | 2.7(2.2-3.2) | 2.5(2.0-3.2) |
| Residence |  |  |  |  |  |  |  |  |  |  |  |
| Urban | 2036 | 12.9(11.5-14.3) | 1.0 |  | 1059 | 6.2(5.3-7.1) | 1.0 |  | 279 | 1.7(1.3-2.0) | 1.0 |
| Rural | 1774 | 17.7(15.1-20.2) | 1.4(1.2-1.8) |  | 982 | 9.2(7.4-11.0) | 1.5(1.2-2.0) |  | 270 | 2.1(1.6-2.6) | 1.3(0.9-1.8) |
| Region |  |  |  |  |  |  |  |  |  |  |  |
| North | 462 | 9.9(8.2-11.7) | 1.0 |  | 262 | 5.1(3.4-6.7) | 1.0 |  | 65 | 1.2(0.6-1.7) | 1.0 |
| Northeast | 502 | 12.5(9.9-15.2) | 1.3(1-1.8) |  | 275 | 6.7(4.7-8.7) | 1.4(0.8-2.2) |  | 78 | 1.7(0.8-2.7) | 1.5(0.7-3.1) |
| Northwest | 401 | 15.5(11.5-19.4) | 1.7(1.2-2.4) |  | 261 | 9.3(5.8-12.7) | 1.9(1.1-3.3) |  | 66 | 2.4(1.6-3.2) | 2.0(1.1-3.7) |
| Central | 460 | 17.1(11.1-23.2) | 1.9(1.2-3) |  | 266 | 9.4(6.2-12.6) | 1.9(1.2-3.2) |  | 88 | 3.4(1.9-4.8) | 2.9(1.5-5.7) |
| East | 769 | 17.6(14.0-21.2) | 1.9(1.4-2.6) |  | 384 | 8.1(6.8-9.3) | 1.6(1.1-2.4) |  | 92 | 1.8(1.0-2.5) | 1.5(0.8-2.9) |
| South | 459 | 15.4(11.2-19.5) | 1.6(1.1-2.4) |  | 217 | 7.8(4.4-11.2) | 1.6(0.9-2.8) |  | 55 | 1.5(0.9-2.1) | 1.3(0.7-2.5) |
| Southwest | 757 | 19.9(15.1-24.8) | 2.3(1.6-3.3) |  | 376 | 10.2(6.9-13.4) | 2.1(1.3-3.5) |  | 105 | 2.6(1.5-3.8) | 2.3(1.2-4.4) |
| Grade |  |  |  |  |  |  |  |  |  |  |  |
| 10 (15–< 17 y) | 1113 | 15.5(11.1-19.9) | 1.0 |  | 577 | 8.1(4.7-11.5) | 1.0 |  | 159 | 1.8(1.3-2.4) | 1.0 |
| 11 (16–< 18 y) | 1272 | 15.0(11.9-18.2) | 1(0.6-1.6) |  | 697 | 7.5(4.9-10.1) | 0.9(0.4-1.9) |  | 180 | 1.7(1.2-2.3) | 0.9(0.6-1.6) |
| 12 (17–< 19 y) | 1425 | 17.1(14.8-19.4) | 1.1(0.8-1.5) |  | 767 | 8.6(7.0-10.3) | 1.1(0.7-1.6) |  | 210 | 2.3(1.7-2.9) | 1.3(0.7-2.5) |
| Cigarette use |  |  |  |  |  |  |  |  |  |  |  |
| No | 3059 | 13.6(12.0-15.3) | 1.0 |  | 1486 | 6.4(5.2-7.6) | 1.0 |  | 341 | 1.3(0.9-1.7) | 1.0 |
| Yes | 751 | 59.6(56.1-63.1) | 9.3(7.9-11) |  | 554 | 40.8(35.8-45.8) | 10.2(7.6-13.6) |  | 208 | 14(10.7-17.2) | 12.3(8-18.8) |
| E-Cigarette use |  |  |  |  |  |  |  |  |  |  |  |
| No | 3322 | 14.6(12.9-16.4) | 1.0 |  | 1660 | 7.1(5.8-8.5) | 1.0 |  | 411 | 1.6(1.2-1.9) | 1.0 |
| Yes | 464 | 55.9(51.4-60.4) | 7.6(6.5-8.8) |  | 365 | 38.6(33.0-44.3) | 8.2(5.7-11.8) |  | 128 | 13.5(9.6-17.5) | 9.9(6.4-15.5) |
| *Academic high school* |  |  |  |  |  |  |  |  |  |  |  |
| Total | 2340 | 13.3(11.1-15.6) |  |  | 1183 | 6.3(5.0-7.6) |  |  | 288 | 1.4(1.0-1.7) |  |
| Gender |  |  |  |  |  |  |  |  |  |  |  |
| Girls | 807 | 10.4(6.2-14.5) | 1.0 |  | 352 | 4.2(1.9-6.5) | 1.0 |  | 84 | 0.8(0.6-1.1) | 1.0 |
| Boys | 1533 | 17.2(14.7-19.7) | 1.8(1.1-2.9) |  | 831 | 8.5(6.8-10.2) | 2.1(1.2-3.9) |  | 204 | 1.9(1.4-2.5) | 2.3(1.8-3.0) |
| Residence |  |  |  |  |  |  |  |  |  |  |  |
| Urban | 1221 | 9.8(8.7-10.9) | 1.0 |  | 605 | 4.6(3.9-5.3) | 1.0 |  | 136 | 1.1(0.8-1.3) | 1.0 |
| Rural | 1119 | 16.3(12.8-19.7) | 1.8(1.3-2.4) |  | 578 | 7.4(5.4-9.5) | 1.7(1.2-2.4) |  | 152 | 1.5(1.0-2.1) | 1.4(0.9-2.3) |
| Region |  |  |  |  |  |  |  |  |  |  |  |
| North | 243 | 8.7(7.4-9.9) | 1.0 |  | 139 | 4.3(2.9-5.8) | 1.0 |  | 28 | 1.0(0.4-1.5) | 1.0 |
| Northeast | 296 | 13.6(10.2-16.9) | 1.7(1.2-2.3) |  | 195 | 8.3(4.9-11.7) | 2.0(1.1-3.5) |  | 49 | 2.2(1.2-3.2) | 2.3(1.1-4.8) |
| Northwest | 260 | 13.2(7.5-19.0) | 1.6(1-2.7) |  | 141 | 6.9(4.1-9.8) | 1.6(0.9-2.9) |  | 38 | 2.0(0.7-3.3) | 2.1(0.9-5.1) |
| Central | 433 | 14.9(10.5-19.4) | 1.8(1.3-2.7) |  | 190 | 6.0(4.7-7.4) | 1.4(0.9-2.2) |  | 38 | 1.1(0.3-1.8) | 1.1(0.4-2.9) |
| East | 343 | 15.5(10.0-21.0) | 1.9(1.2-3.1) |  | 143 | 6.6(3.2-9.9) | 1.6(0.8-3.0) |  | 35 | 1.1(0.8-1.3) | 1.1(0.6-2.0) |
| South | 316 | 9.4(7.0-11.8) | 1.1(0.8-1.5) |  | 161 | 4.7(3.3-6.1) | 1.1(0.7-1.7) |  | 39 | 1.1(0.6-1.5) | 1.1(0.5-2.3) |
| Southwest | 449 | 16.1(8.8-23.4) | 2(1.2-3.6) |  | 214 | 7.7(2.7-12.6) | 1.8(0.8-4.0) |  | 61 | 2.1(0.2-4.0) | 2.2(0.8-6.6) |
| Grade |  |  |  |  |  |  |  |  |  |  |  |
| 10 (15–< 17 y) | 627 | 12.8(7.9-17.6) | 1.0 |  | 309 | 6.0(3.4-8.6) | 1.0 |  | 83 | 1.1(0.7-1.4) | 1.0 |
| 11 (16–< 18 y) | 795 | 13.1(10.2-16.0) | 1.0(0.6-1.8) |  | 400 | 5.7(4.1-7.4) | 1.0(0.5-1.8) |  | 95 | 1.3(0.8-1.9) | 1.3(0.8-2.1) |
| 12 (17–< 19 y) | 918 | 15.3(13.4-17.2) | 1.2(0.8-1.8) |  | 474 | 7.3(5.9-8.6) | 1.2(0.9-1.8) |  | 110 | 1.7(1.2-2.3) | 1.7(1.1-2.6) |
| Cigarette use |  |  |  |  |  |  |  |  |  |  |  |
| No | 1984 | 12.3(10.3-14.3) | 1.0 |  | 915 | 5.2(4.1-6.4) | 1.0 |  | 200 | 1.0(0.7-1.3) | 1.0 |
| Yes | 356 | 54.0(48.5-59.5) | 8.4(6.8-10.5) |  | 267 | 37.1(30-44.2) | 10.7(7.9-14.6) |  | 88 | 11.9(7.2-16.5) | 13.5(8.8-20.7) |
| E-Cigarette use |  |  |  |  |  |  |  |  |  |  |  |
| No | 2110 | 12.9(10.6-15.1) | 1.0 |  | 1011 | 5.7(4.3-7.1) | 1.0 |  | 237 | 1.2(0.8-1.5) | 1.0 |
| Yes | 217 | 50.6(43.5-57.7) | 7.0(5.2-9.4) |  | 165 | 33.7(28.8-38.6) | 8.4(6.2-11.4) |  | 47 | 9.9(5.6-14.2) | 9.4(5.6-15.8) |
| *Vocational high school* |  |  |  |  |  |  |  |  |  |  |  |
| Total | 1470 | 20.5(18.3-22.8) |  |  | 858 | 12.3(10.5-14.1) |  |  | 261 | 3.3(2.3-4.3) |  |
| Gender |  |  |  |  |  |  |  |  |  |  |  |
| Girls | 452 | 13.6(11.0-16.1) | 1.0 |  | 219 | 6.6(5.5-7.7) | 1.0 |  | 70 | 1.8(0.9-2.8) | 1.0 |
| Boys | 1018 | 27.2(24.1-30.3) | 2.4(1.9-3) |  | 639 | 16.8(14.7-19) | 2.9(2.3-3.6) |  | 191 | 4.5(3.4-5.5) | 2.5(1.6-3.8) |
| Residence |  |  |  |  |  |  |  |  |  |  |  |
| Urban | 815 | 21.8(18.6-25.1) | 1.0 |  | 454 | 11.0(8.8-13.1) | 1.0 |  | 143 | 3.3(2.1-4.5) | 1.0 |
| Rural | 655 | 20.7(17.5-23.9) | 0.9(0.7-1.2) |  | 404 | 12.9(10.6-15.3) | 1.2(0.9-1.6) |  | 118 | 3.3(2.0-4.6) | 1.0(0.6-1.7) |
| Region |  |  |  |  |  |  |  |  |  |  |  |
| North | 219 | 16.0(10.9-21.2) | 1.0 |  | 123 | 8.7(4.8-12.6) | 1.0 |  | 37 | 2.3(0.8-3.7) | 1.0 |
| Northeast | 105 | 22.7(19.7-25.8) | 1.5(1-2.3) |  | 66 | 12.9(12.3-13.6) | 1.6(1.0-2.6) |  | 17 | 3.0(2.4-3.7) | 1.4(0.7-2.7) |
| Northwest | 200 | 26.5(17.5-35.4) | 1.9(1-3.4) |  | 125 | 15.1(9.4-20.8) | 1.9(1.0-3.6) |  | 50 | 6.6(3.7-9.6) | 3.1(1.4-7.0) |
| Central | 336 | 24.6(19.3-29.9) | 1.7(1-2.8) |  | 194 | 13.5(8.7-18.2) | 1.6(0.9-3.1) |  | 54 | 3.6(1.5-5.7) | 1.6(0.6-4.0) |
| East | 116 | 15.0(12.4-17.6) | 0.9(0.6-1.4) |  | 74 | 10.2(7.1-13.3) | 1.2(0.7-2.2) |  | 20 | 2.4(0.4-4.4) | 1.1(0.4-3.2) |
| South | 186 | 20.4(14.0-26.8) | 1.4(0.8-2.4) |  | 114 | 11.8(5.2-18.5) | 1.4(0.6-3.2) |  | 39 | 3.3(0.5-6.2) | 1.5(0.5-4.5) |
| Southwest | 308 | 27.1(23.3-31.0) | 2.0(1.3-3.1) |  | 162 | 14.8(11.3-18.4) | 1.8(1.0-3.2) |  | 44 | 3.6(2.4-4.9) | 1.6(0.8-3.5) |
| Grade |  |  |  |  |  |  |  |  |  |  |  |
| 10 (15–< 17 y) | 486 | 21.7(17.8-25.6) | 1.0 |  | 268 | 12.8(7.8-17.7) | 1.0 |  | 76 | 3.5(2.0-5.0) | 1.0 |
| 11 (16–< 18 y) | 477 | 19.8(12.3-27.3) | 0.9(0.5-1.6) |  | 297 | 11.9(4.8-19) | 0.9(0.3-2.6) |  | 85 | 2.7(1.4-4.0) | 0.7(0.4-1.5) |
| 12 (17–< 19 y) | 507 | 21.6(12.2-31.1) | 1.0(0.6-1.6) |  | 293 | 12.1(6.2-18) | 0.9(0.6-1.5) |  | 100 | 3.7(1.3-6.1) | 1.3(0.8-2.2) |
| Cigarette use |  |  |  |  |  |  |  |  |  |  |  |
| No | 1075 | 17.1(15.1-19.1) | 1.0 |  | 571 | 9.3(7.5-11) | 1.0 |  | 141 | 2.1(1.0-3.2) | 1.0 |
| Yes | 395 | 65.1(60.5-69.7) | 8.9(7.2-10.9) |  | 287 | 44.3(38.8-49.9) | 7.8(5.3-11.4) |  | 120 | 15.9(11.7-20.2) | 8.8(4.4-17.7) |
| E-Cigarette use |  |  |  |  |  |  |  |  |  |  |  |
| No | 1212 | 18.9(16.7-21.2) | 1.0 |  | 649 | 10.6(8.6-12.6) | 1.0 |  | 174 | 2.5(1.4-3.6) | 1.0 |
| Yes | 247 | 61.6(55.0-68.2) | 7.1(5.4-9.4) |  | 200 | 43.6(34.6-52.5) | 6.5(3.8-11.1) |  | 81 | 17.2(11.3-23) | 8.0(3.8-17.2) |

Abbreviation: CI, confidence interval.

Monitoring points in counties and county-level cities are defined as rural areas, while monitoring points in districts are defined as urban areas.

| **Table S6: Proportion of middle school students who started drinking alcohol and experienced drunkenness by the age of 13 in China, 2021** | | | | | | | | | | | |
| --- | --- | --- | --- | --- | --- | --- | --- | --- | --- | --- | --- |
|  | **Onset age %(95%CI)** | | | | | | | | | | |
|  | **Drink alcohol** | | | | |  | **Get drunk on alcohol** | | | | |
|  | **Among all students** | |  | **Among lifetime drinkers** | |  | **Among all students** | |  | **Among students who have experienced being drunk** | |
|  | **≤13 years** | **>13 years** |  | **≤13 years** | **>13 years** |  | **≤13 years** | **>13 years** |  | **≤13 years** | **>13 years** |
| Total | 31.2(27.3-35.2) | 4.6(3.4-5.8) |  | 87.2(84.8-89.5) | 12.8(10.5-15.2) |  | 7.1(5.8-8.4) | 2.2(1.4-3.1) |  | 76.2(71.5-80.8) | 23.8(19.2-28.5) |
| Gender |  |  |  |  |  |  |  |  |  |  |  |
| Girls | 26.9(23.2-30.5) | 3.9(2.7-5.1) |  | 87.3(84.4-90.2) | 12.7(9.8-15.7) |  | 5.0(3.9-6.0) | 1.7(0.9-2.4) |  | 74.9(68.9-81.0) | 25.1(19.0-31.1) |
| Boys | 35.1(30.7-39.5) | 5.2(4.0-6.4) |  | 87.1(85.1-89.2) | 12.9(10.8-14.9) |  | 9.1(7.4-10.7) | 2.7(1.8-3.7) |  | 76.8(72.6-81.0) | 23.2(19.0-27.4) |
| Residence |  |  |  |  |  |  |  |  |  |  |  |
| Urban | 28.7(26.7-30.6) | 3.6(3.0-4.2) |  | 88.9(87.2-90.6) | 11.1(9.4-12.8) |  | 5.2(4.3-6.1) | 1.3(1.0-1.6) |  | 79.6(76.3-82.8) | 20.4(17.2-23.7) |
| Rural | 32.8(26.3-39.3) | 5.2(3.3-7.1) |  | 86.3(83.0-89.7) | 13.7(10.3-17.0) |  | 8.3(6.1-10.5) | 2.8(1.4-4.1) |  | 74.9(68.9-81.0) | 25.1(19.0-31.1) |
| Region |  |  |  |  |  |  |  |  |  |  |  |
| Northwest | 16.5(11.8-21.2) | 6.1(2.6-9.5) |  | 73.2(64.3-82.0) | 26.8(18.0-35.7) |  | 4.6(2.6-6.6) | 2.5(0.8-4.1) |  | 65.0(57.2-72.9) | 35.0(27.1-42.8) |
| Northeast | 21.6(15.4-27.9) | 3.9(1.8-6.1) |  | 84.7(78.8-90.7) | 15.3(9.3-21.2) |  | 4.9(3.1-6.8) | 2.1(0.8-3.3) |  | 70.6(61.6-79.7) | 29.4(20.3-38.4) |
| North | 31.1(29.0-33.2) | 2.5(1.7-3.4) |  | 92.5(90.4-94.6) | 7.5(5.4-9.6) |  | 4.0(2.9-5.1) | 0.6(0.3-0.9) |  | 86.8(79.5-94.2) | 13.2(5.8-20.5) |
| Central | 38.7(31.9-45.5) | 4.3(3.6-5.1) |  | 61.7(57.7-65.8) | 38.3(34.2-42.3) |  | 10.3(7.9-12.7) | 2.8(1.8-3.9) |  | 78.4(73.7-83.1) | 21.6(16.9-26.3) |
| East | 33.0(19.7-46.2) | 4.3(2.1-6.5) |  | 88.5(87.0-89.9) | 11.5(10.1-13.0) |  | 7.1(3.6-10.6) | 1.7(0.2-3.3) |  | 80.6(73.6-87.6) | 19.4(12.4-26.4) |
| South | 27.7(22.9-32.5) | 5.0(3.2-6.8) |  | 84.7(79.9-89.6) | 15.3(10.4-20.1) |  | 5.5(4.0-7.0) | 2.0(1.1-2.9) |  | 73.7(67.7-79.7) | 26.3(20.3-32.3) |
| Southwest | 30.8(28.6-33.0) | 6.5(1.3-11.8) |  | 82.5(71.1-93.8) | 17.5(6.2-28.9) |  | 8.7(5.5-11.8) | 3.9(0.4-7.3) |  | 69.2(53.8-84.6) | 30.8(15.4-46.2) |
| Grade |  |  |  |  |  |  |  |  |  |  |  |
| 7 (12–< 14 y) | 28.6(24.1-33.1) | 1.2(0.3-2.1) |  | 96.0(93.5-98.4) | 4.0(1.6-6.5) |  | 7.5(6.1-8.9) | 0.5(0.1-0.9) |  | 93.4(88.9-97.9) | 6.6(2.1-11.1) |
| 8 (13–< 15 y) | 31.9(27.0-36.8) | 2.9(1.6-4.3) |  | 91.6(88.2-95.1) | 8.4(4.9-11.8) |  | 6.8(4.8-8.9) | 1.5(0.8-2.2) |  | 82.2(75.0-89.4) | 17.8(10.6-25.0) |
| 9 (14–< 16 y) | 33.4(29.7-37.0) | 10.0(8.4-11.6) |  | 77.0(74.6-79.3) | 23.0(20.7-25.4) |  | 7.1(5.7-8.4) | 4.8(3.1-6.6) |  | 59.3(53.5-65.2) | 40.7(34.8-46.5) |
| Cigarette use |  |  |  |  |  |  |  |  |  |  |  |
| No | 71.7(64.2-79.3) | 16.4(7.8-25.0) |  | 81.4(72.0-90.8) | 18.6(9.2-28.0) |  | 33.1(28.3-38.0) | 20.2(12.7-27.6) |  | 62.1(52.3-71.9) | 37.9(28.1-47.7) |
| Yes | 30.1(26.4-33.9) | 4.3(3.3-5.2) |  | 87.6(85.7-89.5) | 12.4(10.5-14.3) |  | 6.4(5.3-7.6) | 1.8(1.2-2.3) |  | 78.6(75.1-82.0) | 21.4(18.0-24.9) |
| E-Cigarette use |  |  |  |  |  |  |  |  |  |  |  |
| No | 73.9(65.5-82.4) | 13.7(9.4-18.1) |  | 84.3(79.4-89.3) | 15.7(10.7-20.6) |  | 31.7(27.1-36.3) | 17.7(12.5-22.8) |  | 64.2(57.3-71.1) | 35.8(28.9-42.7) |
| Yes | 30.3(26.5-34.1) | 4.4(3.3-5.4) |  | 87.4(85.2-89.7) | 12.6(10.3-14.8) |  | 6.6(5.4-7.8) | 1.9(1.2-2.5) |  | 77.9(73.6-82.2) | 22.1(17.8-26.4) |

Abbreviation: CI, confidence interval.

Monitoring points in counties and county-level cities are defined as rural areas, while monitoring points in districts are defined as urban areas.

| **Table S7: Proportion of high school students who started drinking alcohol and experienced drunkenness by the age of 13 in China, 2021** | | | | | | | | | | | |
| --- | --- | --- | --- | --- | --- | --- | --- | --- | --- | --- | --- |
|  | **Onset age %(95%CI)** | | | | | | | | | | |
|  | **Drink alcohol** | | | | |  | **Get drunk on alcohol** | | | | |
|  | **Among all students** | |  | **Among lifetime drinkers** | |  | **Among all students** | |  | **Among students who have experienced being drunk** | |
|  | **≤13 years** | **>13 years** |  | **≤13 years** | **>13 years** |  | **≤13 years** | **>13 years** |  | **≤13 years** | **>13years** |
| Total | 31.2(28.1-34.3) | 23.9(22-25.7) |  | 56.7(53.2-60.1) | 43.3(39.9-46.8) |  | 6.4(4.7-8.1) | 9.4(8.4-10.4) |  | 40.4(33.2-47.6) | 59.6(52.4-66.8) |
| Gender |  |  |  |  |  |  |  |  |  |  |  |
| Girls | 25.1(21.3-28.8) | 22.1(19.0-25.2) |  | 53.1(47.3-59.0) | 46.9(41.0-52.7) |  | 5.0(2.1-7.9) | 6.2(5.3-7.1) |  | 44.7(29.2-60.2) | 55.3(39.8-70.8) |
| Boys | 37.1(34.3-40.0) | 25.6(23.7-27.4) |  | 59.2(56.2-62.1) | 40.8(37.9-43.8) |  | 7.8(6.7-8.9) | 12.6(11.3-13.9) |  | 38.1(34.4-41.8) | 61.9(58.2-65.6) |
| Residence |  |  |  |  |  |  |  |  |  |  |  |
| Urban | 28.5(25.8-31.1) | 21.8(20.1-23.4) |  | 56.7(53.2-60.1) | 43.3(39.9-46.8) |  | 4.5(3.8-5.1) | 8.4(7.3-9.5) |  | 34.7(30.6-38.8) | 65.3(61.2-69.4) |
| Rural | 32.9(28.3-37.4) | 25.2(22.3-28.0) |  | 56.7(51.7-61.6) | 43.3(38.4-48.3) |  | 7.6(5.0-10.1) | 10.1(8.6-11.5) |  | 42.9(33.1-52.7) | 57.1(47.3-66.9) |
| Region |  |  |  |  |  |  |  |  |  |  |  |
| Northwest | 21.3(18.0-24.6) | 24.8(21.6-27.9) |  | 46.2(41.6-50.8) | 53.8(49.2-58.4) |  | 5.2(4.3-6.2) | 10.2(6.9-13.6) |  | 33.8(28.0-39.6) | 66.2(60.4-72.0) |
| Northeast | 19.5(15.0-24.0) | 25.1(16.4-33.7) |  | 43.7(38.9-48.5) | 56.3(51.5-61.1) |  | 4.5(3.5-5.6) | 12.6(7.4-17.8) |  | 26.3(20.9-31.7) | 73.7(68.3-79.1) |
| North | 30.0(27.7-32.4) | 17.5(16.1-18.9) |  | 63.2(60.5-65.8) | 36.8(34.2-39.5) |  | 3.8(3.5-4.2) | 6.1(4.5-7.7) |  | 38.7(32.9-44.4) | 61.3(55.6-67.1) |
| Central | 36.1(29.9-42.3) | 26.3(22.0-30.7) |  | 57.8(55.3-60.4) | 42.2(39.6-44.7) |  | 6.9(5.0-8.8) | 10.7(8.9-12.5) |  | 39.1(35.9-42.4) | 60.9(57.6-64.1) |
| East | 34.1(26.4-41.8) | 23.5(20.2-26.9) |  | 59.2(50.7-67.6) | 40.8(32.4-49.3) |  | 7.9(3.0-12.8) | 7.5(6.5-8.5) |  | 51.4(33.2-69.6) | 48.6(30.4-66.8) |
| South | 25.7(19.3-32.2) | 25.8(23.7-28.0) |  | 49.9(44.2-55.6) | 50.1(44.4-55.8) |  | 3.6(2.4-4.8) | 9.0(7.0-10.9) |  | 28.5(22.0-35.1) | 71.5(64.9-78.0) |
| Southwest | 33.8(27.6-39.9) | 23.6(17.8-29.4) |  | 58.9(50.3-67.5) | 41.1(32.5-49.7) |  | 7.9(4.8-11.0) | 12.0(8.0-16.1) |  | 39.7(26.5-52.9) | 60.3(47.1-73.5) |
| Grade |  |  |  |  |  |  |  |  |  |  |  |
| 10 (15–< 17 y) | 34.2(29.4-39.0) | 17.9(16.0-19.9) |  | 65.6(61.0-70.2) | 34.4(29.8-39.0) |  | 8.5(4.1-13.0) | 7.0(6.1-7.8) |  | 55.1(41.5-68.7) | 44.9(31.3-58.5) |
| 11 (16–< 18 y) | 30.1(27.6-32.6) | 24.6(22.2-27.0) |  | 55.0(51.9-58.2) | 45.0(41.9-48.1) |  | 5.4(4.2-6.7) | 9.6(7.5-11.8) |  | 36.1(32.0-40.1) | 63.9(59.9-68.0) |
| 12 (17–< 19 y) | 28.9(25.2-32.5) | 30.1(27.8-32.5) |  | 48.9(45.8-52.0) | 51.1(48.0-54.2) |  | 4.9(4.2-5.6) | 12.2(10.3-14。0) |  | 28.7(25.5-31.8) | 71.3(68.2-74.5) |
| Cigarette use |  |  |  |  |  |  |  |  |  |  |  |
| No | 55.3(50.4-60.3) | 38.4(33.6-43.2) |  | 59.0(54.0-64.1) | 41.0(35.9-46.0) |  | 15.5(13.1-18.0) | 44.0(40.6-47.5) |  | 26.1(22.3-29.9) | 73.9(70.1-77.7) |
| Yes | 30.0(26.8-33.1) | 23.1(21.2-25.0) |  | 56.4(52.7-60.1) | 43.6(39.9-47.3) |  | 5.9(4.2-7.6) | 7.7(6.9-8.5) |  | 43.6(35.2-51.9) | 56.4(48.1-64.8) |
| E-Cigarette use |  |  |  |  |  |  |  |  |  |  |  |
| No | 55.5(48.7-62.2) | 40.3(33.6-47.0) |  | 57.9(50.9-64.9) | 42.1(35.1-49.1) |  | 16.9(14.5-19.2) | 39.0(34.7-43.4) |  | 30.2(26.1-34.3) | 69.8(65.7-73.9) |
| Yes | 30.4(27.1-33.7) | 23.4(21.5-25.3) |  | 56.5(52.7-60.4) | 43.5(39.6-47.3) |  | 6.1(4.3-7.8) | 8.5(7.6-9.5) |  | 41.6(33.6-49.6) | 58.4(50.4-66.4) |
| *Academic high school* |  |  |  |  |  |  |  |  |  |  |  |
| Total | 32.0(29.4-34.7) | 22.1(19.5-24.7) |  | 59.2(55.6-62.8) | 40.8(37.2-44.4) |  | 6.1(4.2-8.1) | 7.6(6.2-8.9) |  | 44.6(35.2-54.0) | 55.4(46.0-64.8) |
| Gender |  |  |  |  |  |  |  |  |  |  |  |
| Girls | 26.8(22.3-31.2) | 20.7(17.1-24.3) |  | 56.4(49.5-63.3) | 43.6(36.7-50.5) |  | 5.5(1.5-9.5) | 4.9(3.8-6.0) |  | 53.0(33.6-72.4) | 47.0(27.6-66.4) |
| Boys | 37.6(35.3-39.9) | 23.6(21.5-25.6) |  | 61.5(59.5-63.5) | 38.5(36.5-40.5) |  | 6.7(5.7-7.8) | 10.4(8.7-12.2) |  | 39.3(36.2-42.3) | 60.7(57.7-63.8) |
| Residence |  |  |  |  |  |  |  |  |  |  |  |
| Urban | 29.6(27.5-31.8) | 19.4(17.8-21.0) |  | 60.4(58.0-62.9) | 39.6(37.1-42.0) |  | 4.0(3.5-4.4) | 5.8(5.0-6.6) |  | 40.7(37.3-44.0) | 59.3(56.0-62.7) |
| Rural | 33.6(29.7-37.6) | 23.9(19.7-28.0) |  | 58.5(53.0-64.0) | 41.5(36.0-47.0) |  | 7.5(4.4-10.6) | 8.7(6.6-10.9) |  | 46.2(33.3-59.0) | 53.8(41.0-66.7) |
| Region |  |  |  |  |  |  |  |  |  |  |  |
| Northwest | 21.2(17.1-25.2) | 24.2(20.3-28.1) |  | 46.7(40.1-53.2) | 53.3(46.8-59.9) |  | 5.1(4.2-6.0) | 8.5(5.5-11.5) |  | 37.5(29.4-45.6) | 62.5(54.4-70.6) |
| Northeast | 19.3(14.7-23.9) | 24.0(15.4-32.7) |  | 44.6(39.3-49.9) | 55.4(50.1-60.7) |  | 3.4(2.7-4.2) | 9.8(4.6-15.0) |  | 26.0(18.2-33.9) | 74.0(66.1-81.8) |
| North | 30.6(28.8-32.4) | 17.5(15.9-19.1) |  | 63.6(60.7-66.5) | 36.4(33.5-39.3) |  | 3.6(3.2-3.9) | 5.1(3.9-6.3) |  | 41.1(34.8-47.5) | 58.9(52.5-65.2) |
| Central | 38.8(31.3-46.3) | 24.0(17.7-30.4) |  | 57.8(55.3-60.4) | 42.2(39.6-44.7) |  | 6.6(4.5-8.7) | 8.4(5.9-10.8) |  | 44.1(40.8-47.4) | 55.9(52.6-59.2) |
| East | 35.7(30.0-41.4) | 21.7(16.1-27.4) |  | 62.2(52.7-71.6) | 37.8(28.4-47.3) |  | 8.9(2.6-15.2) | 6.6(5.0-8.3) |  | 57.3(35.6-79.0) | 42.7(21.0-64.4) |
| South | 29.1(24.4-33.7) | 21.4(17.6-25.2) |  | 57.6(53.3-61.9) | 42.4(38.1-46.7) |  | 3.7(2.6-4.8) | 5.7(4.1-7.3) |  | 39.3(32.2-46.4) | 60.7(53.6-67.8) |
| Southwest | 31.4(26.2-36.7) | 22.5(14.4-30.7) |  | 58.2(47.4-69.1) | 41.8(30.9-52.6) |  | 6.0(3.9-8.1) | 10.1(3.7-16.5) |  | 37.3(22.5-52.2) | 62.7(47.8-77.5) |
| Grade |  |  |  |  |  |  |  |  |  |  |  |
| 10 (15–< 17 y) | 34.1(30.5-37.7) | 15.1(12.1-18.1) |  | 69.3(63.9-74.8) | 30.7(25.2-36.1) |  | 8.3(2.9-13.7) | 4.5(3.3-5.6) |  | 65.0(46.5-83.5) | 35.0(16.5-53.5) |
| 11 (16–< 18 y) | 31.2(28.7-33.6) | 23.1(20.0-26.2) |  | 57.4(54.2-60.7) | 42.6(39.3-45.8) |  | 4.9(4.0-5.8) | 8.2(6.0-10.4) |  | 37.4(33.1-41.7) | 62.6(58.3-66.9) |
| 12 (17–< 19 y) | 30.6(26.9-34.3) | 28.9(26.3-31.4) |  | 51.5(48.0-54.9) | 48.5(45.1-52.0) |  | 4.8(4.0-5.7) | 10.5(9.0-12.0) |  | 31.6(27.3-35.9) | 68.4(64.1-72.7) |
| Cigarette use |  |  |  |  |  |  |  |  |  |  |  |
| No | 58.2(53.0-63.5) | 35.2(29.8-40.6) |  | 62.3(56.8-67.9) | 37.7(32.1-43.2) |  | 13.7(11.3-16.1) | 40.3(35.4-45.2) |  | 25.4(21.4-29.3) | 74.6(70.7-78.6) |
| Yes | 31.1(28.5-33.7) | 21.6(19.0-24.2) |  | 59.0(55.2-62.8) | 41.0(37.2-44.8) |  | 5.8(3.8-7.8) | 6.4(5.3-7.6) |  | 47.6(36.7-58.5) | 52.4(41.5-63.3) |
| E-Cigarette use |  |  |  |  |  |  |  |  |  |  |  |
| No | 62.1(56.3-67.9) | 33.9(27.4-40.4) |  | 64.7(58.2-71.1) | 35.3(28.9-41.8) |  | 15.6(12.4-18.9) | 34.9(28.8-41.1) |  | 30.9(25.3-36.6) | 69.1(63.4-74.7) |
| Yes | 31.4(28.7-34.0) | 21.8(19.3-24.4) |  | 58.9(55.2-62.7) | 41.1(37.3-44.8) |  | 5.9(3.9-7.9) | 7.0(5.7-8.3) |  | 45.7(35.6-55.8) | 54.3(44.2-64.4) |
| *Vocational high school* |  |  |  |  |  |  |  |  |  |  |  |
| Total | 29.2(23.2-35.3) | 28.2(26.1-30.2) |  | 50.9(45.5-56.4) | 49.1(43.6-54.5) |  | 7.1(5.5-8.7) | 14.0(12.1-15.9) |  | 33.8(27.9-39.7) | 66.2(60.3-72.1) |
| Gender |  |  |  |  |  |  |  |  |  |  |  |
| Girls | 20.3(17.2-23.5) | 26.0(21.4-30.7) |  | 43.9(37.9-49.8) | 56.1(50.2-62.1) |  | 3.7(2.5-4.9) | 9.9(7.3-12.4) |  | 27.2(18.2-36.2) | 72.8(63.8-81.8) |
| Boys | 36.2(28.6-43.8) | 29.8(27.1-32.5) |  | 54.8(48.0-61.7) | 45.2(38.3-52.0) |  | 9.9(7.8-12.1) | 17.3(14.7-19.9) |  | 36.5(29.9-43.0) | 63.5(57.0-70.1) |
| Residence |  |  |  |  |  |  |  |  |  |  |  |
| Urban | 25.1(18.9-31.2) | 28.6(24.3-33.0) |  | 46.7(37.6-55.7) | 53.3(44.3-62.4) |  | 5.9(3.8-8.0) | 15.9(14.0-17.8) |  | 27.0(20.2-33.7) | 73.0(66.3-79.8) |
| Rural | 31.3(23.0-39.5) | 27.9(25.8-30.1) |  | 52.8(46.6-59.1) | 47.2(40.9-53.4) |  | 7.7(5.7-9.7) | 13(10.5-15.5) |  | 37.3(29.8-44.9) | 62.7(55.1-70.2) |
| Region |  |  |  |  |  |  |  |  |  |  |  |
| North | 21.6(21.6-21.7) | 26.9(23.8-30.1) |  | 44.6(41.7-47.4) | 55.4(52.6-58.3) |  | 5.7(3.0-8.4) | 17.0(16.6-17.3) |  | 25.3(16.8-33.7) | 74.7(66.3-83.2) |
| Northwest | 19.8(14.4-25.2) | 27.6(18.1-37.1) |  | 41.8(33.2-50.4) | 58.2(49.6-66.8) |  | 7.1(4.9-9.3) | 19.4(12.1-26.7) |  | 26.7(21.2-32.2) | 73.3(67.8-78.8) |
| Northeast | 27.4(21.3-33.5) | 17.5(10.2-24.8) |  | 61.0(52.3-69.8) | 39.0(30.2-47.7) |  | 5.2(3.3-7.1) | 10.9(6.9-14.9) |  | 32.3(23.9-40.6) | 67.7(59.4-76.1) |
| Central | 29.1(23.9-34.3) | 32.4(29.5-35.2) |  | 47.4(43.1-51.7) | 52.6(48.3-56.9) |  | 7.7(5.5-9.8) | 16.9(13.6-20.3) |  | 31.1(27.8-34.5) | 68.9(65.5-72.2) |
| East | 30.9(15.7-46.1) | 27.1(25.3-28.9) |  | 53.3(42.4-64.1) | 46.7(35.9-57.6) |  | 5.9(3.7-8.0) | 9.2(7.1-11.3) |  | 39.0(27.7-50.3) | 61.0(49.7-72.3) |
| South | 17.6(8.1-27.2) | 36.5(31.9-41.2) |  | 32.5(21.4-43.7) | 67.5(56.3-78.6) |  | 3.3(1.3-5.4) | 17.1(12.6-21.6) |  | 16.2(10.9-21.6) | 83.8(78.4-89.1) |
| Southwest | 38.1(29.2-47.0) | 25.6(20.2-30.9) |  | 59.9(50.5-69.2) | 40.1(30.8-49.5) |  | 11.5(6.4-16.5) | 15.7(12.6-18.7) |  | 42.3(28.0-56.5) | 57.7(43.5-72.0) |
| Grade |  |  |  |  |  |  |  |  |  |  |  |
| 10 (15–< 17 y) | 34.4(24.2-44.6) | 24.2(20.1-28.4) |  | 58.7(51.0-66.4) | 41.3(33.6-49.0) |  | 9.1(6.0-12.2) | 12.6(10.3-14.9) |  | 41.9(32.6-51.1) | 58.1(48.9-67.4) |
| 11 (16–< 18 y) | 27.6(22.8-32.4) | 28.2(25.6-30.7) |  | 49.5(44.2-54.7) | 50.5(45.3-55.8) |  | 6.7(3.3-10.1) | 13.1(8.4-17.9) |  | 33.9(25.7-42.0) | 66.1(58.0-74.3) |
| 12 (17–< 19 y) | 24.2(19.9-28.6) | 33.4(28.5-38.2) |  | 42.1(38.3-45.8) | 57.9(54.2-61.7) |  | 5.0(3.2-6.8) | 16.6(8.6-24.6) |  | 23.2(17.3-29.2) | 76.8(70.8-82.7) |
| Cigarette use |  |  |  |  |  |  |  |  |  |  |  |
| No | 52.6(45.8-59.4) | 41.4(35.2-47.7) |  | 55.9(49.1-62.7) | 44.1(37.3-50.9) |  | 17.4(12.3-22.4) | 47.7(42.9-52.5) |  | 26.7(19.7-33.7) | 73.3(66.3-80.3) |
| Yes | 27.0(20.9-33.1) | 26.9(24.6-29.2) |  | 50.1(44.2-56.0) | 49.9(44.0-55.8) |  | 6.2(4.7-7.6) | 10.9(9.1-12.7) |  | 36.2(28.9-43.6) | 63.8(56.4-71.1) |
| E-Cigarette use |  |  |  |  |  |  |  |  |  |  |  |
| No | 48.9(37.4-60.4) | 46.6(35.8-57.5) |  | 51.2(39.5-62.8) | 48.8(37.2-60.5) |  | 18.2(13.0-23.4) | 43.4(36.5-50.3) |  | 29.6(21.6-37.5) | 70.4(62.5-78.4) |
| Yes | 28.0(21.4-34.7) | 27.2(25.0-29.4) |  | 50.8(44.2-57.3) | 49.2(42.7-55.8) |  | 6.6(5.0-8.2) | 12.4(10.5-14.3) |  | 34.7(27.6-41.8) | 65.3(58.2-72.4) |

Abbreviation: CI, confidence interval.

Monitoring points in counties and county-level cities are defined as rural areas, while monitoring points in districts are defined as urban areas.

**Table S8: Types of alcoholic beverages consumed in the past year among middle and high school students in China, 2021**

|  | **Unweighted, No.** | **Weighted % (95% CI)** | **OR(95%CI)** |
| --- | --- | --- | --- |
| **Grape wine** |  |  |  |
| Total | 7027 | 40.1(36.5-45.2) |  |
| Gender |  |  |  |
| Girls | 3058 | 40.8(36.5-45.2) | 1.0 |
| Boys | 3969 | 39.5(35.7-43.3) | 0.9(0.8-1.1) |
| Residence |  |  |  |
| Urban | 3798 | 36.5(33.5-39.5) | 1.0 |
| Rural | 3229 | 41.9(36.5-47.4) | 1.3(1-1.6) |
| Region |  |  |  |
| South | 1111 | 37.9(32.6-43.2) | 1.0 |
| Northwest | 508 | 33.0(27.8-38.2) | 0.8(0.6-1.1) |
| Northeast | 489 | 35.1(26.5-43.6) | 0.9(0.6-1.4) |
| North | 789 | 34.9(32.0-37.8) | 0.9(0.7-1.1) |
| Central | 1984 | 44.5(32.3-56.6) | 1.3(0.8-2.3) |
| East | 940 | 38.6(34.5-42.8) | 1.0(0.8-1.4) |
| Southwest | 1206 | 43.1(36.4-49.7) | 1.2(0.9-1.8) |
| School type |  |  |  |
| Middle school | 3024 | 40.0(34.7-45.2) | 1.0 |
| High school | 4003 | 40.1(36.5-43.7)) | 1.0(0.8-1.2) |
| Academic high school | 3018 | 41.6(37.5-45.6) | 1.0 |
| Vocational high school | 985 | 36.9(30.8-43.0) | 0.8(0.6-1.1) |
| Grade |  |  |  |
| 7 (12–< 14 y) | 855 | 40.9(34.1-47.7) | 1.0 |
| 8 (13–< 15 y) | 997 | 40.3(33.6-47.0) | 1.0(0.8-1.2) |
| 9 (14–< 16 y) | 1172 | 39.1(34.1-44.0) | 0.9(0.8-1.1) |
| 10 (15–< 17 y) | 1313 | 39.8(33.8-45.9) | 1.0(0.7-1.3) |
| 11 (16–< 18 y) | 1343 | 39.4(36.6-42.2) | 0.9(0.7-1.2) |
| 12 (17–< 19 y) | 1347 | 41.2(37.4-45.0) | 1.0(0.8-1.3) |
| **Fruit wine** |  |  |  |
| Total | 7104 | 37.8(34.8-40.9) |  |
| Gender |  |  |  |
| Girls | 3701 | 45.4(41.1-49.6) | 1.0 |
| Boys | 3403 | 32.3(29.9-34.8) | 0.6(0.5-0.6) |
| Residence |  |  |  |
| Urban | 4301 | 42.0(39.2-44.7) | 1.0 |
| Rural | 2803 | 35.7(31.4-39.9) | 0.8(0.6-1) |
| Region |  |  |  |
| South | 1040 | 35.4(31.8-39.0) | 1.0 |
| Northwest | 838 | 54.6(49.4-59.7) | 2.2(1.7-2.8) |
| Northeast | 403 | 31.1(24.9-37.3) | 0.8(0.6-1.1) |
| North | 1067 | 45.9(42.6-49.2) | 1.5(1.3-1.9) |
| Central | 1755 | 37.2(31.8-42.6) | 1.1(0.8-1.4) |
| East | 919 | 35.3(28.7-41.9) | 1.0(0.7-1.4) |
| Southwest | 1082 | 35.4(25.4-45.3) | 1.0(0.6-1.6) |
| School type |  |  |  |
| Middle school | 3045 | 37.6(34.3-40.9) | 1.0 |
| High school | 4059 | 38.0(34.7-41.4) | 1.0(0.9-1.1) |
| Academic high school | 3026 | 39.4(35.4-43.4) | 1.0 |
| Vocational high school | 1033 | 34.8(30.5-39.1) | 0.8(0.7-1.0) |
| Grade |  |  |  |
| 7 (12–< 14 y) | 805 | 35.7(30.3-41.0) | 1.0 |
| 8 (13–< 15 y) | 999 | 37.7(34.3-41.1) | 1.1(0.9-1.3) |
| 9 (14–< 16 y) | 1241 | 39.0(35.0-42.9) | 1.2(0.9-1.4) |
| 10 (15–< 17 y) | 1309 | 36.0(31.2-40.8) | 1.0(0.8-1.3) |
| 11 (16–< 18 y) | 1363 | 38.3(35.0-41.7) | 1.1(0.9-1.3) |
| 12 (17–< 19 y) | 1387 | 39.9(36.6-43.2) | 1.2(1.0-1.5) |
| **Rice wine** |  |  |  |
| Total | 5402 | 32.3(27.2-37.3) |  |
| Gender |  |  |  |
| Girls | 2563 | 36.6(30.5-42.6) | 1.0 |
| Boys | 2839 | 29.1(24.6-33.7) | 0.7(0.6-0.8) |
| Residence |  |  |  |
| Urban | 3244 | 31.0(25.9-36.2) | 1.0 |
| Rural | 2158 | 32.9(25.7-40.1) | 1.1(0.7-1.6) |
| Region |  |  |  |
| South | 609 | 18.8(11.4-26.2) | 1.0 |
| Northwest | 288 | 19.0(11.7-26.2) | 1.0(0.5-2.0) |
| Northeast | 91 | 7.1(4.1-10.1) | 0.3(0.2-0.6) |
| North | 589 | 30.8(22.0-39.5) | 1.9(1.0-3.6) |
| Central | 1539 | 34.4(20.1-48.7) | 2.3(1.0-5.0) |
| East | 1470 | 46.9(37.7-56.1) | 3.8(2.1-7.0) |
| Southwest | 816 | 26.1(20.9-31.3) | 1.5(0.9-2.6) |
| School type |  |  |  |
| Middle school | 2522 | 33.6(27.6-39.5) | 1.0 |
| High school | 2880 | 31.0(26.3-35.7) | 0.9(0.8-1.0) |
| Academic high school | 2301 | 33.7(28.8-38.7) | 1.0 |
| Vocational high school | 579 | 24.8(18.1-31.4) | 0.6(0.5-0.9) |
| Grade |  |  |  |
| 7 (12–< 14 y) | 675 | 33.5(26.3-40.7) | 1.0 |
| 8 (13–< 15 y) | 827 | 33.2(26.4-40.0) | 1.0(0.8-1.3) |
| 9 (14–< 16 y) | 1020 | 33.9(27.8-39.9) | 1.0(0.8-1.2) |
| 10 (15–< 17 y) | 953 | 32.3(25.8-38.8) | 1.0(0.7-1.2) |
| 11 (16–< 18 y) | 1006 | 30.8(25.8-35.9) | 0.9(0.7-1.2) |
| 12 (17–< 19 y) | 921 | 29.8(26.0-33.5) | 0.8(0.7-1.1) |
| **Yellow rice wine** |  |  |  |
| Total | 978 | 7.6(3.6-11.6) |  |
| Gender |  |  |  |
| Girls | 633 | 7.1(3.2-11.0) | 1.0 |
| Boys | 345 | 8.0(3.5-12.4) | 1.1(0.8-1.6) |
| Residence |  |  |  |
| Urban | 408 | 4.2(3.5-4.8) | 1.0 |
| Rural | 570 | 9.4(3.3-15.6) | 2.4(1.2-5) |
| Region |  |  |  |
| South | 233 | 7.1(1.8-12.4) | 1.0 |
| Northwest | 76 | 5.1(3.1-7.2) | 0.7(0.3-1.7) |
| Northeast | 27 | 2.1(1.1-3.0) | 0.3(0.1-0.7) |
| North | 115 | 5.1(4.1-6.1) | 0.7(0.3-1.6) |
| Central | 255 | 6.2(1.6-10.7) | 0.9(0.3-2.6) |
| East | 197 | 15.1(0.6-29.6) | 2.3(0.6-9.3) |
| Southwest | 75 | 2.7(1.9-3.4) | 0.4(0.2-0.8) |
| School type |  |  |  |
| Middle school | 393 | 7.2(2.7-11.8) | 1.0 |
| High school | 585 | 7.9(4.0-11.9) | 1.1(0.7-1.7) |
| Academic high school | 438 | 9.1(4.4-13.8) | 1.0 |
| Vocational high school | 147 | 5.4(2.8-8.0) | 0.6(0.4-0.9) |
| Grade |  |  |  |
| 7 (12–< 14 y) | 89 | 5.6(2.0-9.2) | 1.0 |
| 8 (13–< 15 y) | 132 | 7.4(3.4-11.4) | 1.4(0.9-2.0) |
| 9 (14–< 16 y) | 172 | 8.3(2.4-14.1) | 1.5(1.0-2.4) |
| 10 (15–< 17 y) | 191 | 10.2(4.2-16.3) | 1.9(0.8-4.5) |
| 11 (16–< 18 y) | 198 | 6.5(2.6-10.4) | 1.2(0.8-1.7) |
| 12 (17–< 19 y) | 196 | 6.8(3.0-10.6) | 1.2(0.8-1.9) |
| **High-proof baijiu** |  |  |  |
| Total | 2037 | 11.1(9.9-12.4) |  |
| Gender |  |  |  |
| Girls | 411 | 5.8(4.8-6.8) | 1.0 |
| Boys | 1626 | 15.0(13.1-16.8) | 2.8(2.3-3.5) |
| Residence |  | ) |  |
| Urban | 1138 | 10.8(9.5-12.1) | 1.0 |
| Rural | 899 | 11.3(9.6-13.1) | 1.1(0.8-1.3) |
| Region |  |  |  |
| South | 233 | 7.7(6.3-9.2) | 1.0 |
| Northwest | 241 | 15.3(10.1-20.5) | 2.2(1.4-3.4) |
| Northeast | 163 | 11.9(9.3-14.5) | 1.6(1.2-2.2) |
| North | 363 | 13.1(12.1-14.2) | 1.8(1.4-2.3) |
| Central | 507 | 11.4(9.0-13.9) | 1.5(1.1-2.1) |
| East | 206 | 9.9(7.4-12.3) | 1.3(0.9-1.8) |
| Southwest | 324 | 12.2(8.2-16.2) | 1.7(1.1-2.5) |
| School type |  |  |  |
| Middle school | 702 | 9.9(8.0-11.7) | 1.0 |
| High school | 1335 | 12.4(10.3-14.5) | 1.3(1.0-1.8) |
| Academic high school | 825 | 10.8(8.7-13.0) | 1.0 |
| Vocational high school | 510 | 15.9(12.9-19.0) | 1.6(1.2-2.1) |
| Grade |  |  |  |
| 7 (12–< 14 y) | 184 | 8.7(5.5-11.8) | 1.0 |
| 8 (13–< 15 y) | 237 | 9.6(7.6-11.5) | 1.1(0.8-1.6) |
| 9 (14–< 16 y) | 281 | 11.0(6.8-15.2) | 1.3(0.7-2.4) |
| 10 (15–< 17 y) | 332 | 9.4(7.7-11.1) | 1.1(0.7-1.7) |
| 11 (16–< 18 y) | 465 | 13.2(10.2-16.1) | 1.6(1.1-2.3) |
| 12 (17–< 19 y) | 538 | 14.9(12.1-17.7) | 1.8(1.2-2.7) |
| **Low-proof baijiu** |  |  |  |
| Total | 2203 | 12.5(10.6-14.4) |  |
| Gender |  |  |  |
| Girls | 552 | 7.9(5.0-10.9) | 1.0 |
| Boys | 1651 | 15.7(13.5-17.9) | 2.2(1.4-3.3) |
| Residence |  |  |  |
| Urban | 1222 | 11.7(10.4-13.1) | 1.0 |
| Rural | 981 | 12.9(10.1-15.7) | 1.1(0.8-1.5) |
| Region |  |  |  |
| South | 251 | 8.3(6.5-10.0) | 1.0 |
| Northwest | 247 | 15.7(11.8-19.6) | 2.1(1.4-3.0) |
| Northeast | 193 | 14.1(8.4-19.8) | 1.8(1.1-3.1) |
| North | 378 | 13.7(11.6-15.8) | 1.8(1.3-2.4) |
| Central | 541 | 11.9(9.3-14.5) | 1.5(1.1-2.1) |
| East | 222 | 12.7(6.3-19.0) | 1.6(0.9-3.0) |
| Southwest | 371 | 13.9(10.4-17.5) | 1.8(1.2-2.6) |
| School type |  |  |  |
| Middle school | 736 | 9.8(8.6-10.9) | 1.0 |
| High school | 1467 | 15.1(11.7-18.5) | 1.6(1.3-2.2) |
| Academic high school | 960 | 14.1(10.1-18.1) | 1.0 |
| Vocational high school | 507 | 17.3(12.6-22.0) | 1.3(0.8-2.0) |
| Grade |  |  |  |
| 7 (12–< 14 y) | 161 | 7.5(5.4-9.6) | 1.0 |
| 8 (13–< 15 y) | 243 | 9.4(7.5-11.2) | 1.3(0.9-1.8) |
| 9 (14–< 16 y) | 332 | 11.7(10.5-12.9) | 1.6(1.2-2.2) |
| 10 (15–< 17 y) | 417 | 14.4(7.8-21.0) | 2.1(1.1-4.0) |
| 11 (16–< 18 y) | 472 | 13.2(10.1-16.3) | 1.9(1.5-2.3) |
| 12 (17–< 19 y) | 578 | 17.7(14.5-21.0) | 2.7(2.0-3.5) |
| **Qingke spirit** |  |  |  |
| Total | 611 | 3.3(2.7-3.8) |  |
| Gender |  |  |  |
| Girls | 224 | 2.8(2.3-3.2) | 1.0 |
| Boys | 387 | 3.6(2.8-4.4) | 1.3(1-1.8) |
| Residence |  |  |  |
| Urban | 364 | 3.8(3.1-4.4) | 1.0 |
| Rural | 247 | 3.0(2.3-3.7) | 0.8(0.6-1.1) |
| Region |  |  |  |
| South | 72 | 2.7(1.5-3.8) | 1.0 |
| Northwest | 59 | 4.1(2.3-5.9) | 1.6(0.8-3.0) |
| Northeast | 49 | 4.0(2.1-5.9) | 1.5(0.8-3.0) |
| North | 98 | 4.1(3.3-4.9) | 1.6(1.0-2.6) |
| Central | 164 | 3.3(2.4-4.1) | 1.2(0.7-2.1) |
| East | 64 | 2.5(1.0-4.1) | 1.0(0.4-2.0) |
| Southwest | 105 | 3.7(2.5-4.9) | 1.4(0.8-2.5) |
| School type |  |  |  |
| Middle school | 247 | 3.1(2.3-3.8) | 1.0 |
| High school | 364 | 3.5(2.9-4.0) | 1.1(0.9-1.4) |
| Academic high school | 235 | 3.1(2.6-3.6) | 1.0 |
| Vocational high school | 129 | 4.3(3.1-5.4) | 1.4(1.0-1.9) |
| Grade |  |  |  |
| 7 (12–< 14 y) | 55 | 2.5(1.7-3.3) | 1.0 |
| 8 (13–< 15 y) | 83 | 3.4(2.4-4.3) | 1.3(0.9-1.9) |
| 9 (14–< 16 y) | 109 | 3.2(2.2-4.2) | 1.3(0.9-1.9) |
| 10 (15–< 17 y) | 121 | 3.4(2.5-4.3) | 1.4(1.0-1.8) |
| 11 (16–< 18 y) | 122 | 3.3(2.5-4.1) | 1.3(0.8-2.0) |
| 12 (17–< 19 y) | 121 | 3.7(2.5-4.9) | 1.5(1.0-2.2) |
| **Others** |  |  |  |
| Total | 1291 | 10.4(7.5-13.4) |  |
| Gender |  |  |  |
| Girls | 652 | 12.6(9.3-16.0) | 1.0 |
| Boys | 639 | 8.9(6.1-11.7) | 0.7(0.6-0.8) |
| Residence |  |  |  |
| Urban | 765 | 15.4(12.7-18.1) | 1.0 |
| Rural | 526 | 8.6(4.9-12.3) | 0.5(0.3-0.9) |
| Region |  |  |  |
| South | 322 | 14.2(8.5-20.0) | 1.0 |
| Northwest | 126 | 15.3(9.5-21.2) | 1.1(0.6-2.1) |
| Northeast | 70 | 6.4(1.7-11.1) | 0.4(0.2-1.0) |
| North | 4 | 32.7(0.0-88.3) | 12.4(1.0-61.2) |
| Central | 197 | 5.2(3.0-7.4) | 0.3(0.2-0.6) |
| East | 202 | 9.8(0.3-19.2) | 0.7(0.2-2.1) |
| Southwest | 370 | 17.6(12.0-23.2) | 1.3(0.7-2.4) |
| School type |  |  |  |
| Middle school | 644 | 10.4(7.0-13.8) | 1.0 |
| High school | 647 | 10.4(7.4-13.5) | 1.0(0.8-1.3) |
| Academic high school | 536 | 11.6(8.0-15.2) | 1.0 |
| Vocational high school | 111 | 7.8(4.5-11.2) | 0.7(0.4-0.9) |
| Grade |  |  |  |
| 7 (12–< 14 y) | 177 | 9.9(7.0-12.7) | 1.0 |
| 8 (13–< 15 y) | 202 | 11.6(6.2-17.0) | 1.2(0.7-2.1) |
| 9 (14–< 16 y) | 265 | 9.8(5.2-14.4) | 1.0(0.6-1.6) |
| 10 (15–< 17 y) | 219 | 11.1(7.4-14.8) | 1.1(0.8-1.7) |
| 11 (16–< 18 y) | 239 | 10.7(6.6-14.8) | 1.1(0.7-1.7) |
| 12 (17–< 19 y) | 189 | 9.4(6.6-12.2) | 1.0(0.6-1.4) |

Abbreviation: CI, confidence interval.

Monitoring points in counties and county-level cities are defined as rural areas, while monitoring points in districts are defined as urban areas.

| **Table S9. Frequency of alcohol drinking among middle and high school students who consumed alcoholic beverages in China, 2021** | | | | | |
| --- | --- | --- | --- | --- | --- |
|  | **Drinking frequency** | | | | |
|  | **≥5 days/ week** | **3~4 days/ week** | **1~2 days/ week** | **1~3 days/ month** | **< 1 days/ month** |
| **Beer** |  |  |  |  |  |
| Total | 3.3(2.4-4.2) | 3.0(2.6-3.4) | 7.6(5.6-9.5) | 34.2(32.7-35.6) | 51.9(50.2-53.7) |
| Gender |  |  |  |  |  |
| Girls | 2.5(1.5-3.4) | 1.7(1.1-2.3) | 7.1(3.2-11.0) | 31.7(29.5-33.9) | 57.1(54.0-60.2) |
| Boys | 3.8(2.7-4.8) | 3.7(3.2-4.2) | 7.8(6.4-9.3) | 35.6(34.1-37.0) | 49.1(47.6-50.7) |
| Residence |  |  |  |  |  |
| Urban | 2.4(1.8-2.9) | 3.0(2.3-3.6) | 7.0(5.9-8.1) | 32.2(30.2-34.3) | 55.5(52.6-58.4) |
| Rural | 3.7(2.4-5.0) | 3.0(2.5-3.6) | 7.8(5.1-10.6) | 35.1(33.2-36.9) | 50.3(48.4-52.3) |
| Region |  |  |  |  |  |
| North | 2.1(0.9-3.2) | 2.1(1.2-3.0) | 7.5(5.3-9.8) | 30.9(26.7-35.1) | 57.4(50.1-64.7) |
| Northeast | 2.8(1.0-4.6) | 2.8(1.6-4.0) | 7.5(6.1-8.9) | 38.7(31.0-46.3) | 48.3(40.0-56.6) |
| Northwest | 3.6(2.3-4.9) | 3.1(1.4-4.8) | 8.2(4.9-11.4) | 36.9(31.7-42.1) | 48.2(39.7-56.8) |
| Central | 3.8(2.1-5.5) | 4.0(3.1-5.0) | 8.1(6.4-9.8) | 35.5(32.9-38.0) | 48.6(45.4-51.7) |
| East | 3.5(0.8-6.1) | 2.4(1.5-3.3) | 9.3(3.3-15.3) | 33.0(31.4-34.7) | 51.9(48.5-55.2) |
| South | 2.6(1.6-3.7) | 3.2(1.8-4.6) | 4.9(3.8-6.0) | 32.4(27.7-37.2) | 56.8(52.8-60.9) |
| Southwest | 3.3(2.6-3.9) | 3.0(2.3-3.7) | 5.3(4.3-6.2) | 35.1(30.4-39.7) | 53.4(49.7-57.1) |
| School type |  |  |  |  |  |
| Middle school | 3.8(2.6-5.0) | 3.2(2.5-3.9) | 8.8(6.6-11.0) | 33.3(31.8-34.8) | 50.9(48.8-53.0) |
| High school | 2.9(2.1-3.7) | 2.9(2.5-3.3) | 6.6(3.6-9.7) | 34.8(32.5-37.1) | 52.7(50.1-55.3) |
| Academic high school | 2.2(1.5-2.9) | 2.0(1.5-2.6) | 6.4(2.3-10.5) | 31.8(29.7-33.8) | 57.6(54.3-60.9) |
| Vocational high school | 4.3(3.1-5.4) | 4.5(3.6-5.4) | 7.1(5.4-8.8) | 40.8(36.9-44.7) | 43.3(40.1-46.6) |
| Grade |  |  |  |  |  |
| 7 (12–< 14 y) | 5.5(3.4-7.6) | 2.9(1.8-4.0) | 6.9(5.2-8.6) | 31.2(28.0-34.4) | 53.5(48.2-58.8) |
| 8 (13–< 15 y) | 2.9(1.2-4.5) | 3.3(2.4-4.1) | 8.0(5.9-10.0) | 33.0(29.7-36.4) | 52.9(48.9-56.8) |
| 9 (14–< 16 y) | 3.5(1.7-5.4) | 3.2(2.0-4.5) | 10.5(7.4-13.7) | 34.7(32.4-37.0) | 48.0(44.5-51.4) |
| 10 (15–< 17 y) | 2.7(1.7-3.6) | 3.6(2.7-4.4) | 9.3(2.6-16.0) | 33.1(27.3-38.9) | 51.4(46.3-56.5) |
| 11 (16–< 18 y) | 2.9(2.2-3.6) | 2.2(1.3-3.0) | 5.0(3.3-6.8) | 37.1(34.9-39.3) | 52.8(50.2-55.3) |
| 12 (17–< 19 y) | 3.1(1.7-4.6) | 2.9(1.6-4.2) | 5.5(4.5-6.4) | 34.5(31.0-37.9) | 54.1(50.6-57.6) |
| Cigarette use |  |  |  |  |  |
| No | 2.8(1.9-3.7) | 2.3(1.8-2.9) | 6.9(4.8-9.0) | 33.3(31.9-34.7) | 54.6(53.1-56.1) |
| Yes | 7.4(5.6-9.3) | 8.9(6.1-11.7) | 13.2(10.2-16.1) | 41.6(37.5-45.7) | 28.9(23.0-34.8) |
| E-Cigarette use |  |  |  |  |  |
| No | 2.8(2.0-3.7) | 2.6(2.0-3.1) | 7.1(4.9-9.2) | 33.8(32.6-35.1) | 53.7(52.0-55.3) |
| Yes | 8.8(6.9-10.8) | 9.3(5.4-13.1) | 13.8(10.7-16.9) | 38.2(33.6-42.8) | 29.9(24.0-35.9) |
| **Wine** |  |  |  |  |  |
| Total | 2.5(1.9-3.1) | 1.5(1.1-1.9) | 4.1(3.2-5.0) | 27.9(24.8-31.0) | 64.0(61.1-66.8) |
| Gender |  |  |  |  |  |
| Girls | 1.7(1.0-2.3) | 1.2(0.6-1.8) | 2.7(2.1-3.3) | 27.2(22.2-32.2) | 67.2(62.3-72.1) |
| Boys | 3.2(2.3-4.1) | 1.8(1.1-2.5) | 5.2(3.9-6.6) | 28.4(25.0-31.8) | 61.4(59.1-63.7) |
| Residence |  |  |  |  |  |
| Urban | 2.4(1.7-3.2) | 1.5(1.1-1.9) | 4.3(3.1-5.6) | 24.7(23.1-26.3) | 67.0(64.4-69.7) |
| Rural | 2.6(1.7-3.4) | 1.6(1.0-2.1) | 4.0(2.9-5.1) | 29.5(25.2-33.7) | 62.4(58.4-66.3) |
| Region |  |  |  |  |  |
| North | 2.2(0.6-3.7) | 1.6(0.5-2.7) | 5.3(2.2-8.4) | 24.7(22.6-26.8) | 66.2(61.5-70.9) |
| Northeast | 4.1(0.8-7.3) | 3.3(2.4-4.2) | 4.6(1.6-7.7) | 23.8(16.4-31.3) | 64.2(56.2-72.3) |
| Northwest | 3.9(2.0-5.8) | 2.6(0.9-4.2) | 3.6(1.8-5.3) | 31.1(26.2-35.9) | 58.9(52.2-65.5) |
| Central | 2.3(1.2-3.4) | 1.7(1.3-2.2) | 4.6(3.1-6.2) | 28.9(25.6-32.1) | 62.5(59.2-65.8) |
| East | 2.5(0.7-4.2) | 1.6(0.2-3.0) | 3.9(1.4-6.5) | 29.3(18.6-40.1) | 62.6(52.5-72.8) |
| South | 2.5(0.8-4.2) | 1.2(0.7-1.7) | 3.2(2.1-4.3) | 22.5(19.9-25.1) | 70.7(66.0-75.3) |
| Southwest | 2.6(2.0-3.3) | 0.8(0.5-1.2) | 3.6(2.0-5.1) | 29.3(23.4-35.2) | 63.7(59.2-68.1) |
| School type |  |  |  |  |  |
| Middle school | 3.0(1.9-4.0) | 1.9(1.2-2.6) | 5.2(4.1-6.2) | 28.7(25.0-32.4) | 61.3(58.4-64.2) |
| High school | 2.2(1.5-2.8) | 1.2(0.5-2.0) | 3.2(2.0-4.3) | 27.1(22.2-32.0) | 66.3(61.2-71.4) |
| Academic high school | 1.9(1.1-2.6) | 0.9(0.6-1.2) | 2.1(1.3-2.9) | 25.4(19.6-31.2) | 69.8(64.7-74.8) |
| Vocational high School | 3.0(1.6-4.5) | 2.2(0.0-5.0) | 6.2(3.2-9.1) | 31.9(25.4-38.4) | 56.7(48.8-64.7) |
| Grade |  |  |  |  |  |
| 7 (12–< 14 y) | 4.6(2.7-6.5) | 2.5(1.2-3.8) | 6.4(3.3-9.5) | 30.3(24.8-35.9) | 56.2(49.5-62.8) |
| 8 (13–< 15 y) | 2.2(0.9-3.4) | 1.5(0.7-2.3) | 6.1(4.0-8.2) | 24.9(20.9-28.9) | 65.3(62.1-68.5) |
| 9 (14–< 16 y) | 2.5(1.2-3.8) | 1.8(0.6-3.0) | 3.6(1.9-5.4) | 30.7(24.3-37.0) | 61.5(55.9-67.0) |
| 10 (15–< 17 y) | 2.1(0.6-3.5) | 2.2(0.3-4.1) | 2.9(1.1-4.8) | 31.3(19.7-42.8) | 61.5(47.1-75.9) |
| 11 (16–< 18 y) | 2.1(0.9-3.3) | 0.6(0.3-0.9) | 2.7(1.8-3.7) | 26.2(20.9-31.5) | 68.4(63.4-73.4) |
| 12 (17–< 19 y) | 2.3(1.5-3.1) | 0.8(0.2-1.4) | 3.8(0.2-7.4) | 23.5(19.8-27.2) | 69.6(64.7-74.4) |
| Cigarette use |  |  |  |  |  |
| No | 2.2(1.6-2.9) | 1.3(0.9-1.7) | 3.7(2.9-4.5) | 27.1(24.2-30.0) | 65.7(62.7-68.7) |
| Yes | 5.9(3.6-8.3) | 4.2(2.2-6.3) | 8.7(5.1-12.3) | 36.4(29.8-43.1) | 44.7(39.4-50.0) |
| E-Cigarette use |  |  |  |  |  |
| No | 2.0(1.5-2.5) | 1.4(0.9-1.8) | 3.9(3.0-4.9) | 27.3(24.2-30.3) | 65.5(62.4-68.5) |
| Yes | 9.5(5.7-13.3) | 4.1(2.1-6.0) | 6.9(4.7-9.1) | 37.6(29.3-45.9) | 42.0(35.9-48.0) |
| **High-proof baijiu** |  |  |  |  |  |
| Total | 5.3(3.1-7.4) | 3.0(0.8-5.1) | 6.2(4.1-8.4) | 30.1(27.2-33.0) | 55.4(51.3-59.6) |
| Gender |  |  |  |  |  |
| Girls | 3.9(1.1-6.7) | 1.5(0.5-2.6) | 4.7(2.5-7.0) | 30.5(24.8-36.2) | 59.4(53.5-65.3) |
| Boys | 5.7(3.3-8.1) | 3.4(0.8-6.0) | 6.6(4.0-9.3) | 30.0(26.8-33.1) | 54.3(49.3-59.3) |
| Residence |  |  |  |  |  |
| Urban | 3.8(2.2-5.4) | 3.2(1.9-4.5) | 7.1(4.7-9.5) | 28.9(26.0-31.9) | 57.0(53.0-61.0) |
| Rural | 6.1(2.9-9.3) | 2.9(0.0-6.1) | 5.8(2.8-8.8) | 30.7(26.6-34.7) | 54.6(48.7-60.6) |
| Region |  |  |  |  |  |
| North | 1.4(0.0-3.4) | 1.4(0.0-3.0) | 9.4(4.4-14.4) | 27.3(23.4-31.2) | 60.5(53.9-67.0) |
| Northeast | 6.4(1.0-11.8) | 5.2(2.0-8.4) | 4.2(1.4-7.0) | 32.2(24.2-40.3) | 52.0(45.8-58.1) |
| Northwest | 6.3(1.4-11.2) | 2.2(1.1-3.3) | 8.2(4.3-12.1) | 38.3(31.2-45.3) | 45.1(41.5-48.7) |
| Central | 4.0(1.2-6.8) | 2.7(0.8-4.5) | 7.4(4.5-10.2) | 31.5(24.5-38.6) | 54.4(46.6-62.2) |
| East | 6.7(0.1-13.2) | 4.9(0.0-13.9) | 4.1(0.0-12.0) | 24.8(21.7-27.9) | 59.5(46.6-72.5) |
| South | 6.2(2.8-9.7) | 2.0(0.0-4.4) | 6.8(1.7-11.9) | 25.8(20.5-31.1) | 59.2(53.3-65.0) |
| Southwest | 6.8(0.5-13.2) | 2.5(0.9-4.0) | 4.3(2.1-6.6) | 33.9(25.3-42.5) | 52.5(42.9-62.0) |
| School type |  |  |  |  |  |
| Middle school | 5.9(1.7-10.1) | 0.8(0.1-1.5) | 5.0(2.9-7.1) | 29.8(25.5-34.0) | 58.5(51.9-65.1) |
| High school | 4.8(3.1-6.5) | 4.6(1.1-8.0) | 7.1(4.1-10.2) | 30.3(26.9-33.7) | 53.2(48.8-57.5) |
| Academic high school | 3.7(1.7-5.6) | 2.3(1.1-3.6) | 4.1(1.8-6.4) | 26.3(22.4-30.2) | 63.6(59.2-68.0) |
| Vocational high school | 6.8(3.2-10.3) | 8.2(0.6-15.7) | 12.0(4.9-19.2) | 36.7(28.1-45.3) | 36.4(30.7-42.0) |
| Grade |  |  |  |  |  |
| 7 (12–< 14 y) | 8.0(2.7-13.2) | 0.5(0.0-1.6) | 5.5(1.1-9.9) | 23.4(13.9-33.0) | 62.6(51.9-73.2) |
| 8 (13–< 15 y) | 7.0(3.4-10.6) | 2.3(0.1-4.4) | 5.2(2.1-8.2) | 25.0(18.7-31.3) | 60.6(56.0-65.2) |
| 9 (14–< 16 y) | 4.0(0.0-9.0) | - | 4.6(1.5-7.7) | 36.4(27.3-45.5) | 55.0(40.8-69.2) |
| 10 (15–< 17 y) | 3.2(0.5-5.8) | 3.2(1.2-5.1) | 9.9(0.0-19.7) | 30.1(21.7-38.5) | 53.7(44.4-63.0) |
| 11 (16–< 18 y) | 6.2(3.2-9.1) | 2.4(0.7-4.1) | 4.8(2.6-7.0) | 31.9(26.7-37.2) | 54.7(48.8-60.6) |
| 12 (17–< 19 y) | 4.8(2.2-7.4) | 7.4(0.0-14.7) | 7.4(3.5-11.2) | 29.0(22.3-35.8) | 51.5(43.1-59.8) |
| Cigarette use |  |  |  |  |  |
| No | 4.8(2.6-7.0) | 3.0(0.2-5.9) | 5.3(2.7-8.0) | 26.6(23.7-29.5) | 60.3(55.5-65.1) |
| Yes | 6.9(3.0-10.8) | 2.8(1.2-4.4) | 9.0(6.4-11.6) | 40.8(32.9-48.6) | 40.6(34.6-46.6) |
| E-Cigarette use |  |  |  |  |  |
| No | 3.9(1.9-5.9) | 2.8(0.6-5.1) | 5.2(2.8-7.7) | 28.9(26.4-31.3) | 59.2(55.3-63.1) |
| Yes | 11.3(5.7-16.8) | 3.9(1.6-6.1) | 11.0(6.5-15.5) | 35.4(26.8-44.0) | 38.5(31.3-45.6) |
| **Low-proof baijiu** |  |  |  |  |  |
| Total | 3.8(2.6-4.9) | 3.0(0.9-5.0) | 4.2(3.2-5.3) | 29.2(26.7-31.7) | 59.9(56.2-63.5) |
| Gender |  |  |  |  |  |
| Girls | 3.4(1.7-5.1) | 1.5(0.2-2.8) | 3.3(1.1-5.6) | 20.8(14.6-27.1) | 70.9(63.7-78.0) |
| Boys | 3.9(2.6-5.2) | 3.5(0.9-6.1) | 4.5(3.6-5.4) | 32.3(28.5-36.0) | 55.8(51.8-59.9) |
| Residence |  |  |  |  |  |
| Urban | 3.5(2.1-4.8) | 2.0(1.1-2.9) | 5.9(4.4-7.5) | 28.6(24.9-32.3) | 60.0(55.4-64.6) |
| Rural | 4.0(2.4-5.5) | 3.4(0.3-6.5) | 3.4(2.1-4.7) | 29.5(26.2-32.7) | 59.8(54.8-64.7) |
| Region |  |  |  |  |  |
| North | 2.2(0.4-4.0) | 1.8(0.2-3.4) | 6.1(4.3-7.9) | 23.7(17.1-30.4) | 66.2(57.7-74.7) |
| Northeast | 5.0(0.0-10.3) | 4.4(1.5-7.4) | 6.1(2.0-10.1) | 28.9(25.9-31.9) | 55.6(51.1-60.1) |
| Northwest | 4.1(1.2-6.9) | 3.1(0.5-5.8) | 7.5(2.6-12.3) | 36.5(30.0-42.9) | 48.9(42.5-55.3) |
| Central | 3.5(1.2-5.8) | 3.4(2.2-4.6) | 6.5(4.2-8.8) | 34.4(28.6-40.3) | 52.1(45.5-58.7) |
| East | 4.0(1.2-6.7) | 3.8(0.0-11.8) | 0.3(0.0-0.6) | 21.8(18.3-25.2) | 70.2(61.1-79.3) |
| South | 3.1(0.0-6.2) | 2.3(0.0-4.6) | 3.8(2.0-5.6) | 31.6(24.2-38.9) | 59.2(50.2-68.3) |
| Southwest | 4.8(2.2-7.4) | 1.7(1.0-2.4) | 4.1(2.0-6.1) | 31.9(26.6-37.3) | 57.5(52.1-62.8) |
| School type |  |  |  |  |  |
| Middle school | 5.6(2.2-9.0) | 3.1(1.7-4.6) | 4.3(2.8-5.8) | 29.9(23.5-36.2) | 57.1(48.9-65.3) |
| High school | 2.7(1.6-3.9) | 2.8(0.1-5.6) | 4.2(2.7-5.6) | 28.8(24.9-32.6) | 61.5(55.9-67.1) |
| Academic high school | 1.8(1.0-2.6) | 0.8(0.2-1.5) | 3.2(1.7-4.7) | 22.0(16.2-27.8) | 72.2(65.3-79.0) |
| Vocational high School | 4.5(1.8-7.2) | 6.8(0.0-13.5) | 6.1(3.5-8.7) | 42.1(33.0-51.2) | 40.6(36.1-45.1) |
| Grade |  |  |  |  |  |
| 7 (12–< 14 y) | 9.5(3.6-15.4) | 3.3(0.0-7.3) | 7.0(1.4-12.6) | 24.5(17.5-31.4) | 55.7(45.2-66.3) |
| 8 (13–< 15 y) | 5.4(1.3-9.5) | 2.8(0.0-5.6) | 4.8(1.5-8.0) | 32.4(23.4-41.5) | 54.6(49.0-60.2) |
| 9 (14–< 16 y) | 4.1(0.7-7.5) | 3.3(1.3-5.2) | 2.8(0.9-4.7) | 30.5(20.1-41.0) | 59.3(46.9-71.6) |
| 10 (15–< 17 y) | 1.5(0.1-2.9) | 2.0(0.2-3.8) | 3.4(0.7-6.1) | 27.8(21.2-34.4) | 65.3(55.6-74.9) |
| 11 (16–< 18 y) | 2.8(1.0-4.6) | 1.4(0.2-2.6) | 3.8(2.0-5.7) | 29.8(25.0-34.5) | 62.3(57.2-67.3) |
| 12 (17–< 19 y) | 3.8(2.0-5.6) | 4.7(0.0-11.2) | 5.1(3.0-7.3) | 29.0(22.1-35.8) | 57.4(52.3-62.5) |
| Cigarette use |  |  |  |  |  |
| No | 3.5(2.1-4.8) | 2.8(0.2-5.5) | 3.3(2.3-4.4) | 25.7(22.5-28.9) | 64.7(60.6-68.8) |
| Yes | 5.1(2.2-7.9) | 3.4(1.1-5.8) | 7.5(5.1-9.9) | 42.4(35.5-49.4) | 41.5(36.0-47.1) |
| E-Cigarette use |  |  |  |  |  |
| No | 2.6(1.8-3.4) | 2.7(0.2-5.1) | 3.3(2.4-4.1) | 27.7(24.8-30.6) | 63.7(59.9-67.5) |
| Yes | 9.9(5.7-14.1) | 4.9(2.3-7.5) | 9.5(5.7-13.4) | 38.0(31.4-44.6) | 37.7(32.8-42.5) |

Abbreviation: CI, confidence interval.

Monitoring points in counties and county-level cities are defined as rural areas, while monitoring points in districts are defined as urban areas.

**Table S10. Typical emotions, occasions, and locations associated with alcohol use among middle and high school students in China, 2021**

|  | Total | | |  | Urban | | |  | Rural | | |
| --- | --- | --- | --- | --- | --- | --- | --- | --- | --- | --- | --- |
|  | Both | Boys | Girls |  | Both | Boys | Girls |  | Both | Boys | Girls |
| **Total** |  |  |  |  |  |  |  |  |  |  |  |
| Emotion |  |  |  |  |  |  |  |  |  |  |  |
| Sadness | 23.6(22.0-25.1) | 23.0(21.6-24.4) | 24.3(22.2-26.5) |  | 23.9(22.6-25.2) | 23.3(21.8-24.7) | 24.7(22.6-26.7) |  | 23.4(21.2-25.6) | 22.9(20.9-24.8) | 24.2(21.0-27.3) |
| Joy | 31.3(29.9-32.6) | 32.5(31.0-33.9) | 29.5(27.7-31.3) |  | 31.5(30.1-32.8) | 32.8(31.3-34.4) | 29.7(27.7-31.6) |  | 31.2(29.2-33.1) | 32.3(30.3-34.3) | 29.4(26.9-31.9) |
| Anxiety | 11.1(10.3-11.9) | 11.1(10.4-11.8) | 11.1(9.7-12.5) |  | 12.2(11.5-12.9) | 11.7(10.8-12.5) | 12.9(11.7-14.2) |  | 10.5(9.4-11.7) | 10.8(9.8-11.8) | 10.1(8.1-12.0) |
| Loneliness | 11.5(10.9-12.2) | 12.7(11.9-13.5) | 9.8(8.2-11.4) |  | 11.7(10.9-12.4) | 12.7(11.7-13.7) | 10.2(9.2-11.3) |  | 11.5(10.5-12.4) | 12.7(11.6-13.8) | 9.6(7.1-12.0) |
| Anger | 4.5(4.1-4.9) | 5.0(4.5-5.5) | 3.8(3.2-4.4) |  | 5.1(4.6-5.7) | 5.5(4.7-6.2) | 4.7(4.0-5.5) |  | 4.2(3.7-4.7) | 4.8(4.1-5.5) | 3.3(2.5-4.1) |
| Daily habit | 5.2(4.7-5.7) | 6.2(5.5-6.8) | 3.7(3.1-4.4) |  | 4.9(4.4-5.4) | 5.4(4.6-6.1) | 4.2(3.7-4.8) |  | 5.4(4.6-6.1) | 6.6(5.7-7.5) | 3.5(2.5-4.5) |
| Drinking without emotional motivation, Passive drinking | 36.0(34.7-37.3) | 36.6(34.7-38.6) | 35.0(33.2-36.9) |  | 33.9(32.8-35.1) | 34.3(32.9-35.7) | 33.5(31.6-35.3) |  | 37.0(35.2-38.8) | 37.8(35.0-40.6) | 35.9(33.3-38.5) |
| Other emotions | 19.1(17.7-20.4) | 16.2(15.1-17.3) | 23.2(21.1-25.3) |  | 21.1(19.7-22.6) | 18.8(17.1-20.4) | 24.3(22.4-26.3) |  | 18.0(16.0-20.0) | 14.9(13.5-16.4) | 22.6(19.5-25.7) |
| Occasion |  |  |  |  |  |  |  |  |  |  |  |
| Gathering with friends or classmates | 45.3(42.7-47.8) | 48.2(45.4-51.0) | 41.0(38.0-44.1) |  | 45.1(42.1-48.0) | 48.4(45.2-51.6) | 40.6(37.5-43.7) |  | 45.4(41.8-49.0) | 48.1(44.2-51.9) | 41.3(36.9-45.6) |
| Family gathering | 51.0(48.1-53.9) | 49.5(46.2-52.8) | 53.3(50.5-56.2) |  | 52.4(50.0-54.8) | 49.9(47.5-52.3) | 55.8(53.2-58.5) |  | 50.3(46.1-54.6) | 49.3(44.5-54.0) | 51.9(47.7-56.2) |
| Banquet, including wedding and funeral | 30.4(27.0-33.9) | 34.0(31.0-36.9) | 25.2(20.7-29.7) |  | 27.0(24.8-29.1) | 30.3(28.3-32.3) | 22.4(19.7-25.1) |  | 32.2(27.2-37.2) | 35.7(31.5-39.9) | 26.7(20.0-33.4) |
| Drink alone | 16.7(15.7-17.8) | 16.2(14.8-17.6) | 17.6(15.9-19.3) |  | 17.6(16.4-18.8) | 16.9(15.5-18.2) | 18.6(17.0-20.3) |  | 16.3(14.9-17.7) | 15.8(13.9-17.8) | 17.0(14.6-19.5) |
| Other occasions | 7.1(6.3-7.9) | 5.9(4.8-6.9) | 8.8(7.7-9.9) |  | 7.4(6.7-8.0) | 6.7(5.8-7.7) | 8.2(7.1-9.4) |  | 6.9(5.7-8.1) | 5.4(4.0-6.9) | 9.1(7.5-10.7) |
| Location |  |  |  |  |  |  |  |  |  |  |  |
| Home or someone else's home | 68.9(67.1-70.7) | 68.2(66.2-70.2) | 69.9(67.9-71.9) |  | 66.4(64.4-68.4) | 65.8(63.6-67.9) | 67.4(64.8-69.9) |  | 70.1(67.7-72.6) | 69.4(66.6-72.2) | 71.3(68.6-74.0) |
| Hotel | 9.0(7.7-10.2) | 11.7(9.8-13.5) | 4.8(4.1-5.6) |  | 7.1(6.3-8.0) | 9.5(8.2-10.7) | 3.9(3.1-4.6) |  | 9.9(8.1-11.7) | 12.7(10.1-15.2) | 5.4(4.4-6.4) |
| Bar | 6.2(5.2-7.2) | 7.5(6.3-8.7) | 4.2(3.3-5.2) |  | 7.4(6.1-8.7) | 8.4(7.1-9.8) | 6.0(4.5-7.4) |  | 5.6(4.2-7.0) | 7.1(5.4-8.7) | 3.3(2.0-4.5) |
| Restaurant | 28.5(25.4-31.6) | 30.8(27.9-33.6) | 24.9(20.6-29.2) |  | 31.9(29.3-34.6) | 34.7(31.8-37.5) | 28.1(25.0-31.2) |  | 26.7(22.2-31.2) | 28.9(24.9-33.0) | 23.2(16.7-29.7) |
| KTV or music bar | 13.1(11.6-14.6) | 13.0(11.4-14.6) | 13.3(10.3-16.2) |  | 14.6(12.8-16.4) | 14.5(12.4-16.5) | 14.8(12.8-16.7) |  | 12.4(10.2-14.5) | 12.3(10.1-14.5) | 12.4(8.0-16.9) |
| Other places | 6.4(5.7-7.1) | 6.1(5.3-6.9) | 6.9(5.9-7.9) |  | 5.2(4.5-5.9) | 5.6(4.9-6.4) | 4.7(3.7-5.7) |  | 7.0(6.0-8.0) | 6.3(5.2-7.4) | 8.1(6.7-9.6) |
| **Middle school students** |  |  |  |  |  |  |  |  |  |  |  |
| Emotion |  |  |  |  |  |  |  |  |  |  |  |
| Sadness | 21.0(18.4-23.5) | 19.1(17.2-21.0) | 23.8(19.7-27.9) |  | 20.4(18.2-22.7) | 18.3(15.9-20.7) | 23.3(20.3-26.3) |  | 21.2(17.6-24.9) | 19.4(16.9-21.9) | 24.0(17.9-30.1) |
| Joy | 25.9(23.6-28.2) | 27.7(25.2-30.1) | 23.2(20.7-25.7) |  | 24.4(22.5-26.2) | 26.0(24.0-28.0) | 22.1(19.5-24.7) |  | 26.6(23.3-29.9) | 28.4(25.0-31.9) | 23.8(20.3-27.3) |
| Anxiety | 9.5(8.7-10.3) | 8.5(7.1-9.8) | 11.0(9.4-12.7) |  | 10.5(9.3-11.6) | 9.1(8.1-10.0) | 12.4(10.3-14.5) |  | 9.0(8.0-10.1) | 8.2(6.3-10.1) | 10.3(8.1-12.6) |
| Loneliness | 10.8(10.0-11.7) | 11.5(10.1-12.9) | 9.8(9.0-10.7) |  | 11.0(9.9-12.0) | 10.5(9.5-11.5) | 11.6(9.6-13.5) |  | 10.8(9.6-11.9) | 11.9(9.9-13.9) | 8.9(8.2-9.6) |
| Anger | 4.5(3.7-5.4) | 4.8(3.9-5.7) | 4.1(3.0-5.2) |  | 5.0(4.1-5.9) | 5.1(3.9-6.3) | 4.9(3.6-6.3) |  | 4.3(3.1-5.5) | 4.7(3.5-5.9) | 3.7(2.2-5.3) |
| Daily habit | 4.8(4.1-5.5) | 5.5(4.7-6.3) | 3.7(2.9-4.6) |  | 4.1(3.4-4.8) | 4.3(3.5-5.1) | 3.8(2.8-4.8) |  | 5.1(4.2-6.0) | 6.1(5.0-7.1) | 3.7(2.5-4.9) |
| Drinking without emotional motivation, Passive drinking | 37.1(34.5-39.7) | 36.8(34.2-39.3) | 37.5(34.2-40.9) |  | 35.2(33.5-36.9) | 34.4(32.6-36.3) | 36.3(33.3-39.4) |  | 38.0(34.2-41.8) | 37.9(34.2-41.6) | 38.2(33.3-43.0) |
| Other emotions | 20.2(17.6-22.8) | 18.7(15.6-21.8) | 22.4(20.2-24.7) |  | 24.5(22.4-26.7) | 23.4(20.8-26.0) | 26.1(23.4-28.9) |  | 18.0(14.4-21.6) | 16.5(12.1-20.8) | 20.5(17.5-23.4) |
| Occasion |  |  |  |  |  |  |  |  |  |  |  |
| Gathering with friends or classmates | 34.5(31.3-37.8) | 35.6(31.8-39.3) | 33.0(30.1-35.8) |  | 32.8(29.7-35.8) | 34.0(30.7-37.4) | 31.0(27.5-34.4) |  | 35.4(30.9-39.9) | 36.3(31.0-41.5) | 34.0(30.1-37.9) |
| Family gathering | 47.2(43.5-50.9) | 46.4(42.2-50.5) | 48.4(45.0-51.9) |  | 49.5(46.6-52.4) | 47.8(44.4-51.3) | 51.9(48.6-55.2) |  | 46.0(40.8-51.3) | 45.7(39.8-51.5) | 46.6(41.7-51.5) |
| Banquet, including wedding and funeral | 29.3(24.7-33.9) | 31.6(27.7-35.6) | 25.8(19.5-32.0) |  | 25.1(22.9-27.4) | 27.4(25.0-29.8) | 21.9(18.7-25.2) |  | 31.3(24.8-37.9) | 33.6(28.1-39.2) | 27.8(18.6-37.0) |
| Drink alone | 17.0(14.8-19.2) | 16.2(14.1-18.3) | 18.2(15.5-20.9) |  | 17.4(15.7-19.0) | 15.8(14.4-17.3) | 19.6(17.0-22.1) |  | 16.8(13.7-19.9) | 16.4(13.4-19.4) | 17.5(13.7-21.3) |
| Other occasions | 9.0(7.8-10.3) | 7.6(6.5-8.7) | 11.2(9.1-13.3) |  | 10.5(9.2-11.7) | 9.8(7.9-11.7) | 11.4(9.2-13.5) |  | 8.3(6.6-10.0) | 6.6(5.3-7.9) | 11.1(8.0-14.1) |
| Location |  |  |  |  |  |  |  |  |  |  |  |
| Home or someone else's home | 70.1(68.3-72.0) | 69.9(67.4-72.4) | 70.5(68.0-72.9) |  | 67.8(66.0-69.6) | 66.5(64.4-68.7) | 69.6(67.1-72.0) |  | 71.3(68.7-73.8) | 71.5(68.0-75.0) | 70.9(67.4-74.4) |
| Hotel | 6.9(5.2-8.5) | 8.1(6.3-9.9) | 5.1(3.4-6.7) |  | 5.1(4.2-5.9) | 6.2(4.9-7.5) | 3.5(2.7-4.3) |  | 7.7(5.4-10.1) | 8.9(6.4-11.5) | 5.9(3.5-8.3) |
| Bar | 3.6(2.7-4.5) | 3.6(2.6-4.6) | 3.6(2.5-4.8) |  | 4.0(2.9-5.1) | 3.9(2.4-5.3) | 4.2(3.0-5.3) |  | 3.4(2.3-4.6) | 3.5(2.2-4.8) | 3.3(1.7-5.0) |
| Restaurant | 22.5(18.7-26.4) | 23.4(19.2-27.6) | 21.2(17.6-24.8) |  | 26.5(23.5-29.6) | 27.7(24.6-30.9) | 24.8(20.9-28.8) |  | 20.6(15.1-26.1) | 21.4(15.4-27.4) | 19.4(14.2-24.5) |
| KTV or music bar | 8.0(6.7-9.3) | 7.7(6.3-9.0) | 8.4(6.7-10.2) |  | 9.2(7.5-11.0) | 8.0(6.0-9.9) | 11.0(8.8-13.1) |  | 7.4(5.7-9.1) | 7.5(5.8-9.3) | 7.2(4.8-9.5) |
| Other places | 7.5(6.5-8.5) | 6.6(5.1-8.1) | 8.8(6.7-10.9) |  | 6.0(5.1-6.8) | 6.7(5.7-7.7) | 5.0(3.7-6.3) |  | 8.2(6.8-9.7) | 6.6(4.5-8.7) | 10.8(7.7-14.0) |
| **High school students** |  |  |  |  |  |  |  |  |  |  |  |
| Emotion |  |  |  |  |  |  |  |  |  |  |  |
| Sadness | 25.8(24.5-27.0) | 26.4(24.9-28.0) | 24.8(23.2-26.5) |  | 26.7(25.2-28.3) | 27.5(26.1-28.9) | 25.7(22.8-28.7) |  | 25.3(23.6-26.9) | 25.9(23.8-28.0) | 24.3(22.4-26.2) |
| Joy | 35.8(34.6-37.1) | 36.7(35.4-38.0) | 34.6(32.6-36.7) |  | 37.4(35.7-39.1) | 38.7(36.7-40.6) | 35.7(33.3-38.1) |  | 35.1(33.3-36.8) | 35.7(34.1-37.4) | 34.0(31.2-36.9) |
| Anxiety | 12.5(11.4-13.5) | 13.4(12.2-14.7) | 11.1(9.1-13.1) |  | 13.6(12.7-14.6) | 13.9(12.6-15.1) | 13.3(11.8-14.9) |  | 11.9(10.3-13.4) | 13.2(11.4-15.0) | 9.9(7.1-12.7) |
| Loneliness | 12.1(10.7-13.6) | 13.8(12.7-14.9) | 9.8(7.1-12.5) |  | 12.3(11.2-13.3) | 14.6(12.9-16.4) | 9.2(7.9-10.4) |  | 12.1(10.0-14.2) | 13.4(12.0-14.8) | 10.1(6.0-14.2) |
| Anger | 4.5(4.0-5.0) | 5.2(4.7-5.7) | 3.5(2.6-4.4) |  | 5.2(4.5-6.0) | 5.8(4.7-6.8) | 4.6(3.7-5.4) |  | 4.1(3.5-4.7) | 4.9(4.3-5.4) | 3.0(1.7-4.2) |
| Daily habit | 5.5(5.0-6.1) | 6.8(6.0-7.7) | 3.8(2.8-4.7) |  | 5.5(4.6-6.5) | 6.3(5.1-7.5) | 4.6(3.7-5.4) |  | 5.5(4.8-6.2) | 7.1(6.0-8.1) | 3.3(1.9-4.7) |
| Drinking without emotional motivation, Passive drinking | 35.1(33.7-36.5) | 36.5(34.0-39.1) | 33.0(31.0-35.0) |  | 32.9(31.2-34.5) | 34.2(31.8-36.6) | 31.2(29.3-33.0) |  | 36.2(34.3-38.1) | 37.7(34.1-41.3) | 34.0(31.1-37.0) |
| Other emotions | 18.1(16.3-19.9) | 14.0(12.2-15.8) | 23.9(20.8-26.9) |  | 18.3(17.2-19.4) | 14.8(13.5-16.1) | 22.9(20.7-25.1) |  | 18.0(15.3-20.7) | 13.6(11.1-16.1) | 24.4(19.9-28.9) |
| Occasion |  |  |  |  |  |  |  |  |  |  |  |
| Gathering with friends or classmates | 54.4(51.9-57.0) | 59.1(56.2-62.0) | 47.7(44.1-51.2) |  | 55.1(51.8-58.4) | 60.4(57.0-63.8) | 48.1(44.1-52.1) |  | 54.1(50.6-57.6) | 58.4(54.4-62.4) | 47.5(42.4-52.5) |
| Family gathering | 54.3(51.9-56.7) | 52.2(49.6-54.8) | 57.4(54.0-60.7) |  | 54.8(51.9-57.7) | 51.6(48.9-54.3) | 58.9(54.9-62.9) |  | 54.0(50.6-57.4) | 52.4(48.8-56.1) | 56.5(51.7-61.2) |
| Banquet, including wedding and funeral | 31.4(28.5-34.3) | 36.0(32.6-39.4) | 24.7(20.9-28.5) |  | 28.4(25.9-31.0) | 32.8(30.4-35.2) | 22.8(19.4-26.1) |  | 32.9(28.8-37.0) | 37.6(32.8-42.3) | 25.8(20.2-31.5) |
| Drink alone | 16.5(15.3-17.8) | 16.1(14.9-17.4) | 17.1(14.0-20.3) |  | 17.8(16.0-19.7) | 17.7(15.8-19.7) | 17.9(15.5-20.4) |  | 15.9(14.2-17.5) | 15.3(13.8-16.8) | 16.7(11.9-21.5) |
| Other occasions | 5.4(4.6-6.2) | 4.4(3.0-5.7) | 6.9(6.0-7.8) |  | 4.9(4.3-5.5) | 4.2(3.6-4.8) | 5.8(4.6-7.0) |  | 5.6(4.5-6.8) | 4.4(2.4-6.5) | 7.5(6.2-8.7) |
| Location |  |  |  |  |  |  |  |  |  |  |  |
| Home or someone else's home | 67.9(66.0-69.8) | 66.9(64.9-68.8) | 69.5(67.2-71.7) |  | 65.4(62.9-67.8) | 65.2(62.4-67.9) | 65.6(62.4-68.9) |  | 69.2(66.7-71.7) | 67.7(65.2-70.2) | 71.7(68.7-74.6) |
| Hotel | 10.7(9.6-11.7) | 14.6(12.7-16.4) | 4.7(3.7-5.6) |  | 8.7(7.7-9.8) | 12.1(10.5-13.7) | 4.2(3.2-5.1) |  | 11.7(10.3-13.1) | 15.8(13.3-18.3) | 4.9(3.6-6.3) |
| Bar | 8.3(7.0-9.7) | 10.6(8.9-12.4) | 4.7(3.7-5.8) |  | 10.1(8.6-11.5) | 12.0(10.5-13.5) | 7.4(5.4-9.4) |  | 7.4(5.5-9.4) | 10.0(7.5-12.5) | 3.2(2.1-4.4) |
| Restaurant | 33.4(30.7-36.0) | 36.8(34.3-39.3) | 28.0(22.4-33.6) |  | 36.2(33.3-39.1) | 40.2(37.0-43.4) | 30.7(27.3-34.1) |  | 31.9(28.1-35.6) | 35.2(31.8-38.5) | 26.5(17.8-35.1) |
| KTV or music bar | 17.3(15.6-19.1) | 17.4(14.8-20.0) | 17.3(13.3-21.3) |  | 18.8(16.7-20.9) | 19.6(17.3-22.0) | 17.7(14.9-20.5) |  | 16.6(14.1-19.0) | 16.3(12.6-19.9) | 17.0(10.9-23.2) |
| Other places | 5.5(4.8-6.2) | 5.7(4.9-6.4) | 5.3(4.1-6.5) |  | 4.7(3.9-5.5) | 4.8(3.7-6.0) | 4.5(3.3-5.6) |  | 6.0(5.0-6.9) | 6.1(5.1-7.1) | 5.8(4.1-7.5) |
| *Academic high school* |  |  |  |  |  |  |  |  |  |  |  |
| Emotion |  |  |  |  |  |  |  |  |  |  |  |
| Sadness | 23.8(22.7-24.9) | 25.5(23.8-27.2) | 21.7(19.4-23.9) |  | 24.1(22.2-25.9) | 25.9(24.2-27.6) | 21.8(18.7-24.9) |  | 23.7(22.4-25.0) | 25.3(22.9-27.8) | 21.6(18.6-24.6) |
| Joy | 35.9(34.5-37.4) | 36.6(35.1-38.1) | 35.1(32.5-37.6) |  | 36.5(34.7-38.2) | 37.1(35.3-38.8) | 35.7(32.6-38.9) |  | 35.7(33.6-37.7) | 36.4(34.3-38.5) | 34.7(31.1-38.4) |
| Anxiety | 10.7(9.8-11.6) | 11.9(10.9-12.9) | 9.2(7.3-11.1) |  | 12.2(10.9-13.5) | 12.9(11.3-14.5) | 11.4(9.5-13.4) |  | 9.9(8.6-11.1) | 11.3(10.1-12.6) | 8.0(5.4-10.6) |
| Loneliness | 11.4(9.8-13.1) | 12.7(11.5-13.9) | 9.8(6.0-13.6) |  | 11.1(9.9-12.2) | 13.5(11.7-15.3) | 8.1(6.9-9.2) |  | 11.6(9.2-14.1) | 12.3(10.8-13.8) | 10.8(5.1-16.5) |
| Anger | 3.9(3.3-4.5) | 4.7(4.0-5.4) | 2.8(1.9-3.7) |  | 4.3(3.7-4.9) | 5.2(4.2-6.2) | 3.2(2.5-3.9) |  | 3.6(2.8-4.5) | 4.5(3.6-5.4) | 2.6(1.3-3.9) |
| Daily habit | 4.8(4.3-5.4) | 6.0(5.1-6.8) | 3.4(2.7-4.2) |  | 5.0(4.1-6.0) | 5.7(4.4-6.9) | 4.3(3.2-5.3) |  | 4.7(4.1-5.4) | 6.1(5.0-7.3) | 3.0(2.0-4.0) |
| Drinking without emotional motivation, Passive drinking | 34.6(33.4-35.9) | 36.0(34.6-37.4) | 32.9(30.4-35.4) |  | 33.3(31.4-35.3) | 35.2(32.8-37.7) | 31.0(29.1-32.9) |  | 35.3(33.6-37.0) | 36.4(34.7-38.2) | 34.0(30.1-37.8) |
| Other emotions | 20.4(18.0-22.8) | 16.2(14.7-17.6) | 25.7(22.2-29.2) |  | 20.3(19.1-21.6) | 16.4(15.0-17.8) | 25.2(23.3-27.1) |  | 20.4(16.7-24.1) | 16.0(14.0-18.1) | 26.0(20.6-31.3) |
| Occasion |  |  |  |  |  |  |  |  |  |  |  |
| Gathering with friends or classmates | 51.1(48.2-53.9) | 56.4(53.7-59.0) | 44.1(40.2-48.1) |  | 50.1(47.2-53.1) | 56.0(52.3-59.6) | 42.9(40.1-45.7) |  | 51.6(47.4-55.7) | 56.6(53.0-60.2) | 44.8(38.9-50.8) |
| Family gathering | 57.2(54.5-60.0) | 53.8(50.7-56.9) | 61.7(58.4-65.1) |  | 58.3(56.1-60.5) | 54.3(51.7-56.9) | 63.2(60.4-66.0) |  | 56.7(52.6-60.7) | 53.5(48.9-58.2) | 60.8(55.7-66.0) |
| Banquet, including wedding and funeral | 30.0(27.1-32.9) | 34.6(31.4-37.8) | 24.1(20.1-28.0) |  | 27.5(24.5-30.5) | 31.9(28.8-34.9) | 22.2(18.5-25.9) |  | 31.4(27.1-35.7) | 36.1(31.3-40.8) | 25.1(19.2-31.1) |
| Drink alone | 16.9(14.6-19.3) | 16.8(15.1-18.6) | 17.1(12.9-21.2) |  | 18.0(15.7-20.3) | 18.1(15.7-20.5) | 17.9(14.8-21.1) |  | 16.3(12.9-19.7) | 16.2(13.8-18.5) | 16.5(10.1-22.9) |
| Other occasions | 5.3(4.3-6.4) | 4.8(3.4-6.1) | 6.1(5.2-7.0) |  | 5.3(4.6-6.0) | 4.9(4.0-5.8) | 5.9(4.7-7.1) |  | 5.3(3.8-6.9) | 4.7(2.7-6.8) | 6.2(4.9-7.4) |
| Location |  |  |  |  |  |  |  |  |  |  |  |
| Home or someone else's home | 69.4(67.2-71.5) | 68.8(66.7-70.9) | 70.1(67.4-72.9) |  | 66.9(64.6-69.2) | 66.0(63.1-68.8) | 68.2(65.1-71.3) |  | 70.7(67.6-73.8) | 70.3(67.5-73.2) | 71.2(67.4-75.1) |
| Hotel | 9.5(8.3-10.6) | 12.9(10.5-15.4) | 4.6(3.9-5.4) |  | 7.5(6.3-8.7) | 10.3(8.5-12.1) | 3.9(2.8-5.1) |  | 10.5(9.0-12.1) | 14.4(10.7-18.1) | 5.1(4.2-6.0) |
| Bar | 7.2(5.8-8.5) | 9.5(7.6-11.5) | 3.9(2.8-4.9) |  | 8.0(6.7-9.4) | 9.6(7.8-11.4) | 6.0(4.0-7.9) |  | 6.7(4.7-8.6) | 9.5(6.8-12.3) | 2.6(1.5-3.8) |
| Restaurant | 33.2(29.6-36.8) | 36.4(33.9-38.9) | 28.9(21.8-35.9) |  | 35.7(32.7-38.6) | 40.8(37.2-44.4) | 29.0(25.4-32.6) |  | 31.9(26.6-37.2) | 34.1(30.8-37.4) | 28.8(17.8-39.7) |
| KTV or music bar | 16.8(14.2-19.5) | 17.3(14.8-19.8) | 16.3(10.2-22.4) |  | 16.0(14.1-17.9) | 16.9(14.2-19.5) | 14.8(12.6-17.1) |  | 17.3(13.3-21.3) | 17.5(13.9-21.0) | 17.1(7.7-26.5) |
| Other places | 5.2(4.6-5.7) | 5.1(4.2-6.0) | 5.3(4.2-6.3) |  | 4.7(3.8-5.6) | 4.8(3.7-5.9) | 4.5(3.4-5.6) |  | 5.4(4.6-6.2) | 5.2(4.0-6.4) | 5.7(4.3-7.1) |
| *Vocational high school* |  |  |  |  |  |  |  |  |  |  |  |
| Emotion |  |  |  |  |  |  |  |  |  |  |  |
| Sadness | 30.1(27.1-33.2) | 28.2(25.1-31.3) | 33.6(27.6-39.6) |  | 33.5(31.6-35.4) | 31.1(28.4-33.8) | 37.1(34.9-39.4) |  | 28.6(24.7-32.5) | 27.0(23.0-31.0) | 31.7(22.8-40.7) |
| Joy | 35.6(32.1-39.2) | 36.9(33.3-40.4) | 33.4(28.8-38.0) |  | 39.7(35.8-43.6) | 42.4(38.2-46.6) | 35.7(31.3-40.1) |  | 33.8(29.2-38.3) | 34.6(30.5-38.7) | 32.1(25.3-38.9) |
| Anxiety | 16.4(14.4-18.4) | 16.4(13.3-19.5) | 16.4(12.6-20.2) |  | 17.2(15.1-19.3) | 16.2(13.7-18.7) | 18.8(14.6-23.0) |  | 16.0(13.4-18.7) | 16.5(12.3-20.7) | 15.1(9.8-20.4) |
| Loneliness | 13.7(12.0-15.4) | 16.0(13.9-18.0) | 9.6(7.4-11.9) |  | 15.3(12.0-18.6) | 17.2(12.4-22.1) | 12.3(10.1-14.5) |  | 13.0(11.0-15.1) | 15.5(13.3-17.6) | 8.2(5.0-11.4) |
| Anger | 5.9(5.0-6.7) | 6.0(4.8-7.2) | 5.6(4.2-6.9) |  | 7.6(5.4-9.8) | 7.0(4.4-9.6) | 8.6(6.1-11.0) |  | 5.0(4.2-5.9) | 5.6(4.3-6.9) | 4.0(2.1-5.8) |
| Daily habit | 7.1(5.6-8.5) | 8.4(7.0-9.9) | 4.6(2.3-7.0) |  | 6.8(4.3-9.3) | 7.7(4.9-10.6) | 5.4(3.1-7.7) |  | 7.2(5.5-9.0) | 8.7(7.1-10.4) | 4.2(0.8-7.7) |
| Drinking without emotional motivation, Passive drinking | 36.1(32.1-40.0) | 37.6(31.3-43.8) | 33.3(30.4-36.2) |  | 31.7(27.7-35.6) | 31.7(26.6-36.7) | 31.7(25.5-37.8) |  | 38.0(32.9-43.2) | 40.0(31.7-48.3) | 34.2(31.2-37.2) |
| Other emotions | 13.0(11.3-14.7) | 9.8(7.2-12.5) | 18.7(15.4-22.1) |  | 13.2(11.3-15.0) | 11.2(8.2-14.2) | 16.2(12.9-19.4) |  | 12.9(10.6-15.2) | 9.3(5.8-12.8) | 20.1(15.4-24.8) |
| Occasion |  |  |  |  |  |  |  |  |  |  |  |
| Gathering with friends or classmates | 61.8(56.8-66.7) | 64.2(56.7-71.7) | 57.3(51.7-62.8) |  | 67.7(64.6-70.9) | 70.8(66.5-75.2) | 63.0(59.6-66.4) |  | 59.1(52.8-65.4) | 61.6(52.1-71.0) | 54.2(46.1-62.3) |
| Family gathering | 47.9(44.9-50.9) | 49.1(44.6-53.6) | 45.7(42.4-49.0) |  | 45.9(41.6-50.3) | 45.5(40.7-50.2) | 46.6(40.7-52.5) |  | 48.8(45.1-52.4) | 50.6(45.1-56.0) | 45.2(41.1-49.2) |
| Banquet, including wedding and funeral | 34.3(29.8-38.8) | 38.7(34.8-42.5) | 26.4(20.6-32.3) |  | 30.7(27.9-33.6) | 34.9(31.6-38.2) | 24.4(20.4-28.4) |  | 35.9(29.9-42.0) | 40.2(35.4-44.9) | 27.5(18.7-36.4) |
| Drink alone | 15.7(11.9-19.4) | 14.7(11.5-18.0) | 17.4(11.8-23.0) |  | 17.4(14.6-20.1) | 17.0(13.3-20.7) | 18.0(15.2-20.8) |  | 14.9(9.6-20.2) | 13.8(9.7-17.9) | 17.1(8.6-25.6) |
| Other occasions | 5.5(4.5-6.5) | 3.6(1.7-5.5) | 9.0(5.9-12.2) |  | 3.8(2.4-5.2) | 2.6(1.3-3.9) | 5.6(2.5-8.7) |  | 6.3(5.3-7.3) | 4.0(1.4-6.5) | 10.8(6.7-15.0) |
| Location |  |  |  |  |  |  |  |  |  |  |  |
| Home or someone else's home | 64.8(62.6-67.0) | 63.3(61.0-65.7) | 67.7(64.2-71.3) |  | 61.6(58.0-65.3) | 63.4(58.5-68.3) | 58.8(55.2-62.5) |  | 66.2(63.4-69.0) | 63.3(60.6-66.1) | 72.7(69.1-76.4) |
| Hotel | 13.2(11.8-14.6) | 17.5(15.1-20.0) | 4.7(2.3-7.1) |  | 11.6(9.9-13.3) | 16.0(12.8-19.1) | 4.8(2.0-7.5) |  | 14.0(12.3-15.7) | 18.2(15.0-21.3) | 4.6(1.2-8.1) |
| Bar | 10.8(8.1-13.5) | 12.6(7.9-17.4) | 7.0(5.2-8.9) |  | 14.9(12.5-17.3) | 17.4(14.7-20.0) | 11.0(6.8-15.3) |  | 8.9(5.3-12.5) | 10.8(4.7-16.8) | 4.8(2.7-6.8) |
| Restaurant | 33.6(31.0-36.2) | 37.5(32.9-42.1) | 25.8(21.9-29.6) |  | 37.4(32.2-42.5) | 38.8(33.5-44.2) | 35.1(28.1-42.0) |  | 31.9(28.6-35.2) | 37.0(31.0-43.0) | 20.6(16.9-24.2) |
| KTV or music bar | 18.3(14.0-22.6) | 17.6(12.6-22.5) | 19.9(15.5-24.3) |  | 25.6(22.2-29.0) | 25.8(21.9-29.6) | 25.3(20.6-30.1) |  | 15.1(9.4-20.7) | 14.3(8.3-20.3) | 16.8(9.9-23.8) |
| Other places | 6.3(5.0-7.6) | 6.8(5.5-8.0) | 5.4(3.2-7.6) |  | 4.6(3.0-6.3) | 4.8(2.3-7.3) | 4.3(1.4-7.3) |  | 7.1(5.4-8.7) | 7.5(6.2-8.9) | 6.0(3.1-9.0) |
